# Ultralong organic room-temperature phosphorescence of tetraphenylene *via* lactose functionalization and chromophore isolation

**DOI:** 10.1039/d6sc01359g

**Published:** 2026-04-13

**Authors:** Xin Niu, Mingxing Chen, Yuan Zhang, Guang-jian Liu, Su-Hang Qian, Hui Zhang, Jia-Qing Jiang, Hongwei Tan, Guo-wen Xing

**Affiliations:** a College of Chemistry, Beijing Normal University Beijing 100875 China gwxing@bnu.edu.cn hongwei.tan@bnu.edu.cn; b College of Chemistry and Molecular Engineering, Peking University Beijing 100871 China

## Abstract

Achieving efficient and ultralong room-temperature phosphorescence (RTP) in purely organic systems is challenging. This work addresses this issue by unlocking unprecedented RTP performance in nonplanar tetraphenylene (TeP), a chromophore traditionally limited by aggregation-induced quenching and poor processability. Through an innovative dual strategy of lactose functionalization and supramolecular chromophore isolation within superabsorbent polymers (SAPs), ultralong organic RTP is achieved with lifetimes (*τ*) of 1.27 s (298 K) and 2.21 s (77 K). Serving as a host matrix, the SAPs provide a rigid environment that effectively isolates the chromophores and suppresses non-radiative decay. Concurrently, lactose functionalization confers high aqueous solubility, thereby significantly broadening the scope of practical applications. Furthermore, a high performance supramolecular Förster resonance energy transfer (FRET) system is constructed *via* the co-assembly of fluorescent acceptors with the RTP matrix, enabling long-lived multicolor afterglow (>200 ms, naked-eye visible), redshifted emission (584–753 nm), and ultralarge Stokes shifts (up to 473 nm). These advancements facilitate applications in information encryption and afterglow displays. This work not only reveals the untapped potential of TeP for ultralong RTP but also establishes carbohydrate functionalization as a versatile paradigm for designing next generation, amorphous, and organic RTP materials with specific properties.

## Abstract 2

A water-soluble amorphous nonplanar tetraphenylene system with ultralong room-temperature phosphorescence is achieved *via* lactose functionalization and a core isolation strategy, highlighting its utility in advanced information encryption.

## Graphical abstract

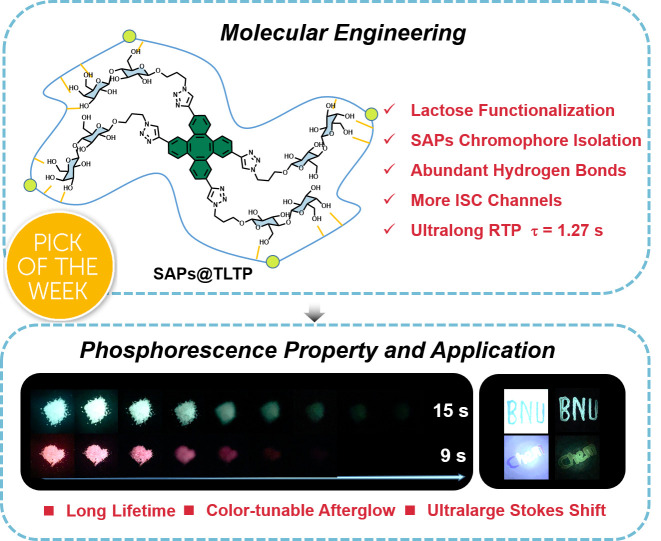

## Introduction

Room-temperature phosphorescence (RTP) materials have attracted much attention because of their long-lived emission, high excitation utilization efficiency, and large Stokes shifts.^1^ Although inorganic, long-persistent luminescent systems incorporating transition metals have been successfully commercialized in industrial applications, their extensive use is limited by inherent limitations such as high cost and environmental toxicity.^2^ In contrast, purely organic RTP materials have distinct advantages, including low cost, excellent biocompatibility, and structural diversity, rendering them excellent candidates for next generation optoelectronic technologies.^3,4^ These organic systems have great potential for applications in organic light-emitting diodes (OLEDs), bioimaging, anticounterfeiting, sensor development and information encryption, *etc.*^5–9^

The sources of organic RTP materials are categorized into single-component phosphorescent systems and multi-component phosphorescent systems.^10,11^ Single-component organic RTP systems typically emit in crystalline form. The precise molecular structure and spatial arrangement within crystals can be determined *via* X-ray diffraction, establishing relationships among molecular structure, aggregate packing modes, and RTP properties.^12–15^ This approach provides valuable guidance for elucidating RTP mechanisms and designing efficient organic RTP materials. However, crystalline phosphors also limit the application of single-component phosphors in practice. When the structure of an organic crystal is disrupted and transitions to an amorphous state, phosphorescence is significantly attenuated or even quenched despite the molecules remaining in the solid state.^16^ Furthermore, most reported single-component organic RTP molecules have planar conjugated structures, and studies on nonplanar single-component organic RTP materials are limited.^17–20^ In 2019, Yuan and coworkers synthesized the spatial conjugation compound AN–MA, obtained its single crystal, and achieved a maximum lifetime of 1.60 s, confirming the feasibility of the spatial conjugation strategy.^17^ In 2023, Yang's group investigated tetraphenylene (TeP) and its halogenated derivatives and revealed that the rigid and highly twisted conformation of TeP suppresses nonradiative RTP pathways and improves electron exchange interactions, facilitating the RTP radiative process.^18^ The crystalline phosphorescence lifetime of the fluorinated derivative TeP–F reached 890 ms, whereas that of unsubstituted TeP was 307 ms. Nonplanar phosphorescent molecules have attracted considerable interest owing to their unique properties and novel design principles. However, these nonplanar molecules emit room-temperature phosphorescence only in their crystalline state, and advancements in this field remain limited by insufficient strategies for improving their phosphorescence performance. With respect to inorganic compounds, nearly all long-lived phosphorescence originates from host–guest doping.^21^ Similarly, in organic RTP systems, multicomponent host–guest doping is still the main method for generating long-lived phosphorescence.^22–24^ The rigid matrix provided by the host material effectively suppresses nonradiative transitions of the guest and isolates triplet excitons from quenching factors such as water and oxygen, improving phosphorescence. Efficient host–guest doping relies on effective intermolecular interactions between the host and the guest. Host materials capable of providing hydrogen-bonding sites and phosphorescent chromophores modified with hydrophilic substituents are typically selected to increase the doping efficiency *via* hydrogen bonding. Hydrophilic substituents often include boric acid, carboxyl, amino, and hydroxyl groups. However, owing to the limited acidity and ionization potential of these functional groups, the water solubility of their derivatives remains low, resulting in suboptimal doping efficiency with hydrophilic host materials.^25^

Our group has recently focused on the lactose functionalization of different chromophores and has demonstrated that this strategy endows chromophores with excellent water solubility.^26–30^ Although significant advances have been made in the study of glycosylated fluorophores,^31–34^ their phosphorescent properties remain systematically unexplored. It is noteworthy that lactose functionalization can modulate the aggregation behaviors, resulting in distinct photophysical properties for the modified systems. Therefore, lactose functionalization has significant potential for the design of novel organic RTP systems. Specifically, the lactose functionalization of existing single-component phosphors exploits the hydrogen-bond formed between lactose units, which serves as a protective matrix to immobilize the chromophore and suppress non-radiative decay. As a result, efficient RTP is achieved even in amorphous solid powders.

**Fig. 1:**
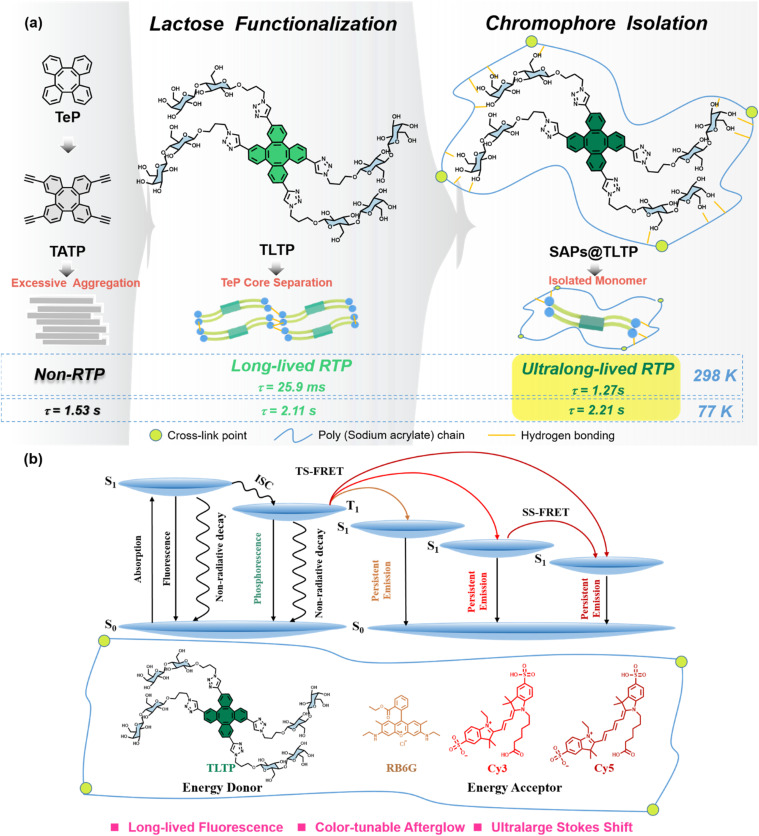
(a) Schematic of the molecular engineering of TATP, TLTP and SAPs@TLTP; (b) FRET systems with color-tunable afterglow emission and Jablonski diagram for illustration of the phosphorescence energy transfer process.

In this work, we selected TeP as the phosphorescent core for molecular engineering (Fig. 1). As a nonplanar, saddle shaped molecule, TeP and its derivatives have been applied in helically chiral macrocycles, asymmetric catalysis, supramolecular assembly and liquid crystal molecular design, *etc.*^35–39^ However, further progress has been hindered by the lack of robust and accessible enantioselective synthetic strategies for structurally diverse chiral TeP derivatives.^40^ We aimed to both expand the application scope of this molecule and provide new insights for organic RTP molecular design. Interestingly, through experimental exploration and theoretical calculations, we discovered that tetraalkynyl tetraphenylene (TATP; Fig. 1a), obtained by modifying TeP with alkynyl groups, exhibited only fluorescence emission at room temperature because of phosphorescence quenching caused by excessive aggregation. Nevertheless, tetra-lactose-functionalized tetraphenylene (TLTP, Fig. 1a), a derivative of TATP, exhibited phosphorescent emission at room temperature with a lifetime of 25.9 ms. We subsequently selected superabsorbent polymers (SAPs), which are composed primarily of sodium polyacrylate as the host matrix, which is a hydrophilic material used in water treatment, coatings, organic glass, and the paper industry. Doping TLTP into the SAPs matrix successfully yielded the amorphous phosphorescent composite SAPs@TLTP, significantly extending the TLTP phosphorescence lifetime to 1.27 s (Fig. 1a). Remarkably, with a FRET-delayed sensitization approach,^41^ we coassembled SAPs@TLTP with commercial fluorophores (rhodamine 6G and cyanine dyes Cy3/Cy5) in a single step (Fig. 1b). This step enabled tunable phosphorescence colors ranging from green to yellow to red, accompanied by a substantial increase in the apparent Stokes shift from 220 nm to 473 nm. Notably, the use of Cy3 as an energy transfer bridge facilitated two-step FRET cascades, substantially increasing the near-infrared phosphorescence efficiency. For the first time, a water-soluble amorphous nonplanar tetraphenylene system with ultralong room-temperature phosphorescence is achieved *via* lactose functionalization and a core isolation strategy.

## Results and discussion

**Fig. 2:**
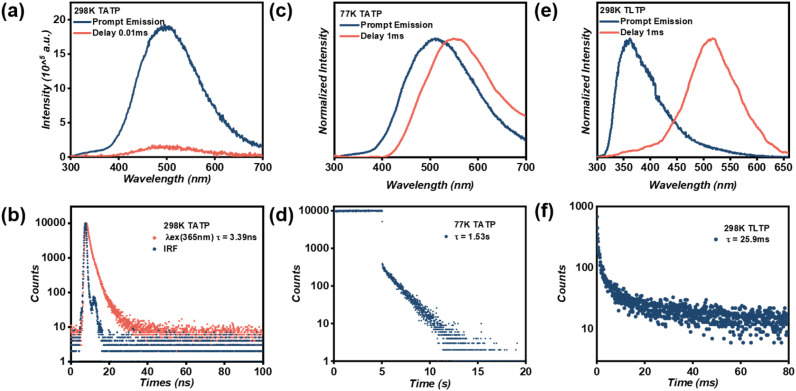
(a) Normalized prompt (blue line) and 0.01 ms-delayed (red line) emission spectra of TATP under 280 nm excitation; (b) fluorescence lifetime decay (measured at 500 nm) profiles of TATP under 365 nm excitation; (c) normalized prompt (blue line) and 1 ms-delayed (red line) emission spectra of TATP under 280 nm excitation at 77 K; (d) phosphorescence lifetime decay (measured at 550 nm) profiles of TATP under 280 nm excitation at 77 K; (e) normalized prompt (blue line) and 1 ms-delayed (red line) emission spectra of TLTP under 280 nm excitation at 298 K; and (f) phosphorescence lifetime decay profiles of TLTP were monitored at 525 nm under 280 nm excitation at 298 K.

Firstly, with 2,7,10,15-tetrabromotetraphenylene (compound 5) as the starting material, the Sonogashira coupling reaction was employed to integrate alkynyl groups into the TeP core, yielding a powdered TATP solid (Scheme S1). Both steady-state photoluminescence (PL) and delayed emission spectra revealed an emission peak at 500 nm for TATP (Fig. 2a). Concurrently, even with a very short gate delay of 0.01 ms, no detectable phosphorescence signal was observed in the delayed spectra, suggesting the absence of phosphorescence emission (Fig. 2a). Lifetime measurements, however, indicated a decay constant of merely 3.39 ns at this wavelength, confirming exclusively fluorescent emission (Fig. 2b). Remarkably, under cryogenic conditions (77 K), the delayed spectra exhibited phosphorescent emission at 550 nm with a lifetime of 1.53 s, revealing its inherent phosphorescence capability (Fig. 1a, 2c and d).

**Fig. 3:**
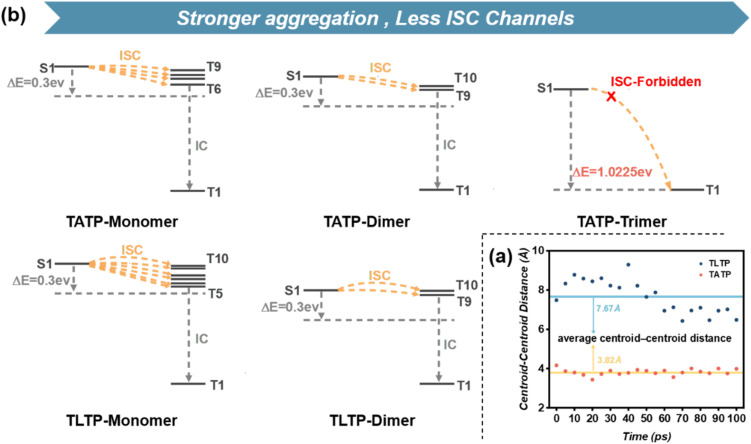
(b) calculated energy levels of the present RTP systems and possible ISC channels are represented by yellow arrows; (a) centroid-centroid distance variation of TLTP-dimers and TATP-dimers in 100 ps through molecular dynamic simulation.

Secondly, lactose units were conjugated to TATP *via* click chemistry, yielding the powdered product TLTP (Scheme S1). This structural modification unlocked the latent phosphorescent potential. While steady-state PL spectra showed only fluorescence, delayed emission spectra (*λ*_ex_ = 280 nm, duration = 1 ms) unambiguously confirmed phosphorescence with a characteristic lifetime of 25.9 ms (Fig. 2e and f). As shown in Fig. 2, TATP exhibits phosphorescence at 77 K (Fig. 2c) but only fluorescence at room temperature (Fig. 2a). In contrast, following the lactose functionalization strategy, TLTP displays distinct RTP (Fig. 2e). This comparison clearly demonstrates that the lactose functionalization strategy effectively activates the phosphorescent potential of TATP, enabling phosphorescence emission at room temperature that was originally observable only at 77 K. This may be attributed to the increased intermolecular separation induced by lactose functionalization, which weakens π–π stacking-driven triplet–triplet annihilation (TTA, T_1_ + T_1_ → S_0_ + S_1_) and thereby facilitates RTP emission. To verify this hypothesis, molecular dynamics simulations were performed on the TLTP and TATP. The results reveal that the average centroid distance within TLTP is 7.67 Å, whereas that in TATP is only 3.82 Å over a 100 ps simulation period (Fig. 3a). This confirms that lactose functionalization indeed increases the intermolecular spacing. Furthermore, weak intermolecular interactions in TATP and TLTP were analyzed using the reduced density gradient (RDG) function, yielding corresponding RDG scatter plots (Fig. S1). The RDG analysis further indicates that TLTP exhibits stronger intermolecular hydrogen-bonding and van der Waals interactions than TATP with these forces originating from the lactose moieties. Thus, the introduction of lactose not only increases the intermolecular distance but also stabilizes this expanded spacing through intermolecular interactions. Previous studies have demonstrated that rigid environments constructed *via* hydrogen-bonding interactions in host matrices can effectively suppress nonradiative transitions and promote RTP.^42–45^ Therefore, we propose that the lactose functionalization strategy adopted in this work similarly contributes to the generation of RTP.

Additionally, to further validate the hypothesis that lactose functionalization increases intermolecular distances, thereby suppressing aggregation and enhancing RTP emission, time-dependent density functional theory (TD-DFT) calculations were performed to evaluate the energy levels of different aggregates of TATP and TLTP (Fig. 3b). Owing to computational limitations, the comparison was confined to monomers, dimers, and trimers. Within the 0.3 eV range of the S1 energy level, monomers of TATP had more Tn states feasible for the intersystem crossing (ISC) process than its dimers did. Notably, the occurrence of ISC is physically impossible in TATP trimers. Similarly, calculations for TLTP molecules revealed the greatest numbers of ISC channels in the monomer, which were significantly reduced in the dimer. Therefore, we conclude that stronger aggregation degrees of TATP and TLTP correlate with less ISC channels, inhibiting efficient RTP emission. Increasing the intermolecular distance can effectively reduce the degree of aggregation, leading to the further conclusion that greater isolation of TLTP molecules promotes easier RTP release.

Furthermore, vibrational analyses were performed on both the TLTP monomer and TLTP dimer. The calculated results (Fig. S2) reveal that the maximum vibrational intensity of the TLTP monomer in the 1000–2000 cm^−1^ region is more than twice that of the TLTP dimer, indicating that the hydrogen-bond network formed by lactose functionalization plays a critical role in reducing non-radiative decay by suppressing molecular vibrations. Meanwhile, we employed root-mean-square deviation (RMSD) analysis (Fig. S3) to evaluate structural differences between the chromophore moieties of TATP and TLTP. The results show that the chromophore cores of TLTP and TATP overlap almost perfectly, with a RMSD value of only 0.025 Å (a RMSD value of 0 Å corresponds to identical structures). This finding demonstrates that the introduction of lactose does not alter the conformation of the chromophore, and consequently does not further influence spin–orbit coupling (SOC). In summary, the room-temperature phosphorescence emission of TLTP can be attributed to two key factors: (i) the increased intermolecular distance effectively suppresses TTA; (ii) the rigid microenvironment provided by the hydrogen-bond network significantly restricts molecular vibrations, thereby suppressing non-radiative decay pathways. To validate this mechanistic hypothesis, we designed an experiment in which 1 wt% TLTP was doped into a SAPs matrix to achieve molecular-level isolation, while simultaneously leveraging the rigid environment provided by SAPs to further suppress molecular vibrations, with the aim of achieving extended phosphorescence lifetime.

**Fig. 4:**
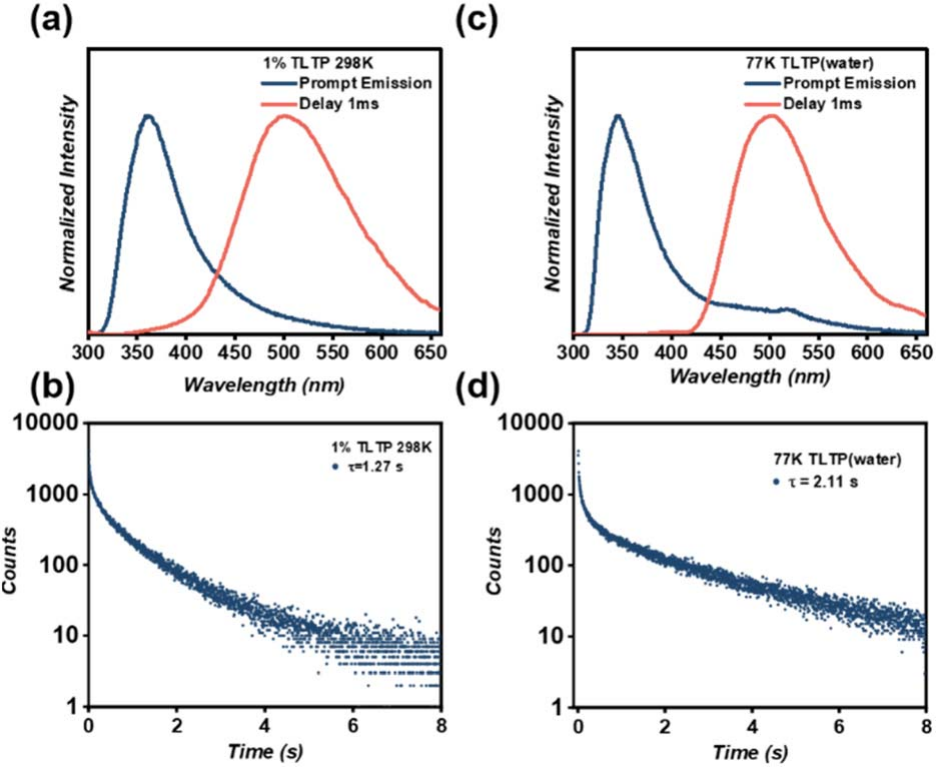
(a) Normalized prompt (blue line) and 1 ms-delayed (red line) emission spectra of SAPs@TLTP under 280 nm excitation at 298 K; (b) phosphorescence lifetime decay profile of SAPs@TLTP was monitored at 500 nm under 280 nm excitation at 298 K; (c) normalized prompt (blue line) and 1 ms-delayed (red line) emission spectra of ultradilute TLTP aqueous solution under 280 nm excitation at 77 K; (d) phosphorescence lifetime decay profile of ultradilute TLTP aqueous solution was monitored at 500 nm under 280 nm excitation at 77 K.

Remarkably, the resulting composite SAPs@TLTP exhibited a phosphorescence lifetime of up to 1.27 s (Fig. 4b), which aligns with our expectations and experimentally supports the conclusion that greater isolation of TLTP molecules facilitates more efficient RTP emission. Moreover, the temperature-dependent photoluminescence spectra reveal that the time-delayed luminescence intensity of SAPs@TLTP progressively decreases with increasing temperature, accompanied by a concomitant reduction in the emission lifetime (Fig. S4). This thermal quenching behavior unambiguously confirms the phosphorescent nature of the 500 nm peak observed in the delayed spectra, and we can observe a 77 K phosphorescence lifetime of 2.21 s (Fig. S4).

In a word, through lactose functionalization, we have transformed TATP, which exhibits only fluorescence at room temperature, into the RTP compound TLTP (*τ* = 25.9 ms). Further employing a chromophore isolation strategy by doping TLTP into SAPs yielded the SAPs@TLTP, which achieved a remarkable 49-fold enhancement in RTP lifetime (*τ* = 1.27 s). Taken together, these dual strategies successfully translate the inherent phosphorescent capability of the TATP chromophore, initially observed only at 77 K, into efficient and ultralong RTP emission at 298 K (Fig. 1,2, S4 and 4). To establish the optimal doping ratio, we varied the TLTP concentration across a range of 0.1 to 5 wt% while maintaining the SAP mass constant at 100 mg. As shown in Fig. S5, with increasing TLTP doping ratio, the phosphorescent emission of SAPs@TLTP redshifted, accompanied by a concomitant reduction in lifetime. A comprehensive evaluation revealed that the system with 1% wt% TLTP exhibited an extended afterglow duration and spectral shift, establishing this optimized composition as the focus of subsequent investigations.

Additionally, stability assessments revealed that the 1 wt% SAPs@TLTP composite exhibited a gradual decrease in both fluorescence intensity and phosphorescence intensity after three weeks of storage under room light-exposed conditions at 25 °C but retained exceptional emission performance, with a phosphorescence lifetime of 1.19 s (Fig. S6). To assign the phosphorescence emission source, we analyzed the delayed spectra of TLTP, SAPs, and SAPs@TLTP. These comparative studies strongly suggest that the 500 nm emission in SAPs@TLTP should originate from aggregated TLTP molecules embedded within the SAP matrix (Fig. S7). Specifically, Fig. S7a demonstrates that the phosphorescence of the SAPs@TLTP composite originates directly from the TLTP guest; and Fig. S7b shows a significant change in the phosphorescence lifetime upon doping, which serves as direct proof of the host–guest interaction and successful incorporation. Remarkably, doping TLTP into SAPs increased the phosphorescence lifetime by 49-fold because of host–guest hydrogen-bonding interactions that rigidify the local environment and suppress nonradiative triplet-state decay.

**Fig. 5:**
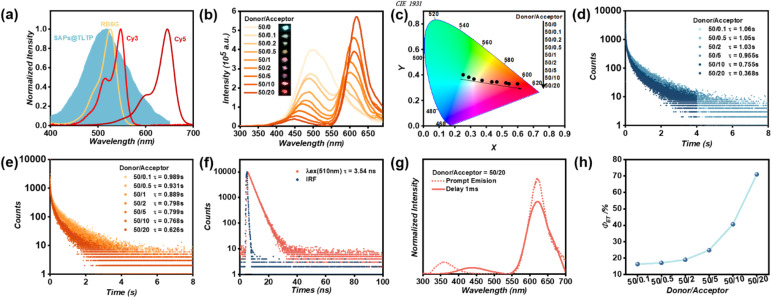
(a) Normalized phosphorescence spectra of SAPs@TLTP under 280 nm excitation and absorption spectra of RB6G, Cy3 and Cy5 under 365 nm excitation; (b) delayed spectra (duration = 1 ms) of SAPs@TLTP with different molar ratios of Cy3 (insert: photograph under 280 nm-UV excitation showing emission color change of SAPs@TLTP itself and with different molar ratios of Cy3) under 280 nm excitation; (c) CIE 1931 diagram showing the color shift in emission for FRET system SAPs@TLTP/Cy3; (d) phosphorescence lifetime decay profiles (measured at 500 nm) of SAPs@TLTP with different molar ratios of Cy3 under 280 nm excitation; (e) phosphorescence lifetime decay profiles (measured at 600 nm) of SAPs@TLTP with different molar ratios of Cy3 under 280 nm excitation; (f) fluorescence lifetime decay profiles of SAPs@TLTP/Cy3 under 510 nm excitation at 600 nm; (g) normalized prompt (dashed line) and 1 ms-delayed (solid line) emission spectra of SAPs@TLTP/Cy3 (the ratio of TLTP/Cy3 is 50/20) under 280 nm excitation; (h) *Φ*_ET_ at different SAPs@TLTP/Cy3 molar ratios.

To construct a FRET system, we selected Cy3 as the energy acceptor for noncovalent conjugation with SAPs@TLTP (denoted as SAPs@TLTP/Cy3). As shown in Fig. 5a, Cy3 has a maximum absorption peak at 548 nm with a broad spectral coverage spanning approximately 450–600 nm, indicating significant overlap with the phosphorescent emission spectra of SAPs@TLTP. The spectra strongly suggest the feasibility of establishing a high efficiency energy transfer system during coassembly. The experimental results indicate that the gated spectra (duration = 1 ms) reveal distinct delayed fluorescence characteristics of Cy3 at 600 nm (Fig. 5b). After the SAPs@TLTP/Cy3 molar ratio gradually increases (*λ*_ex_ = 280 nm), the emission intensity of Cy3 at 600 nm progressively increases with a concomitant redshift of its emission maximum, whereas the phosphorescence intensity of SAPs@TLTP at 500 nm correspondingly attenuates and undergoes a blueshift (Fig. 5b). The observed emission enhancement and red-shift with increasing Cy3 doping ratio are characteristic of J-aggregate formation, where head-to-tail molecular arrangement leads to exciton delocalization and a lower excited-state energy.^46–48^ At a SAPs@TLTP/Cy3 molar ratio of 50 : 20, the delayed fluorescence peak maximum reached 618 nm. This systematic evolution conclusively validates the occurrence of FRET. Furthermore, a macroscopically visible afterglow color gradient (from green to red) after the cessation of UV light corroborated this energy transfer mechanism (Fig. 5b).^49–51^ As shown in Fig. 5c, the CIE 1931 diagram clearly shows a significant redshift in the phosphorescence afterglow of SAPs@TLTP/Cy3. Furthermore, we investigated the optimal doping ratio for the SAPs@TLTP/Cy3 system. As shown in Fig. S8, the red afterglow intensity initially increases and then decreases as the donor/acceptor ratio increases from 50 : 2 to 50 : 60, with the maximum intensity observed at 50 : 20. Therefore, this ratio was determined to be optimal.

The FRET mechanism was further validated *via* time-resolved fluorescence spectroscopy. The SAPs@TLTP exhibited long-lived phosphorescence at 500 nm, with a lifetime of 1.27 s. When the SAPs@TLTP/Cy3 molar ratio was adjusted to 50 : 20, the lifetime decreased significantly to 0.368 s, indicating energy transfer from the triplet state of SAPs@TLTP to the singlet state of Cy3 (Fig. 5d). TRFS analysis revealed that Cy3 excited at 280 nm displayed prolonged emission lifetimes ranging from 0.625 to 0.989 s (Fig. 5e). In contrast, direct excitation of Cy3 at 510 nm yielded only transient fluorescence with a lifetime of 3.54 ns, confirming that the delayed emission originated from a sensitization process (Fig. 5f). As a result, highly overlapping prompt and delayed spectra were observed (Fig. 5g), unequivocally confirming that long-lived fluorescence occurred through the delayed sensitization of the singlets of Cy3 driven by triplet-state exciton-involved energy transfer.

Quantitative analysis of the performance of the FRET system revealed that the energy transfer efficiency (*Φ*_ET_) increased progressively with increasing Cy3 loading ratio, reaching 71.0% at a SAPs@TLTP/Cy3 molar ratio of 50 : 20 (Fig. 5h). Concurrently, the apparent Stokes shift exhibited a marked expansion from 220 nm to 338 nm, highlighting the excellent spectral modulation capability of this FRET system.

Building on the broad emission spectra (380–700 nm) and moderate emission intensity of SAPs@TLTP (Fig. 5a), we further engineered a FRET system by coassembling RB6G with SAPs@TLTP. The occurrence of FRET in SAPs@TLTP/RB6G was unambiguously confirmed by gated spectra (duration = 1 ms) and TRFS analysis (Fig. S9 and S10). Although the *Φ*_ET_ is limited to 28.6% (Fig. S11), as a supplement, the high overlap of the prompt and delayed spectra indicates FRET progress (Fig. S12). As shown in Fig. S13, CIE also definitively exhibited a significant redshift in the phosphorescence afterglow of SAPs@TLTP/RB6G.

**Fig. 6:**
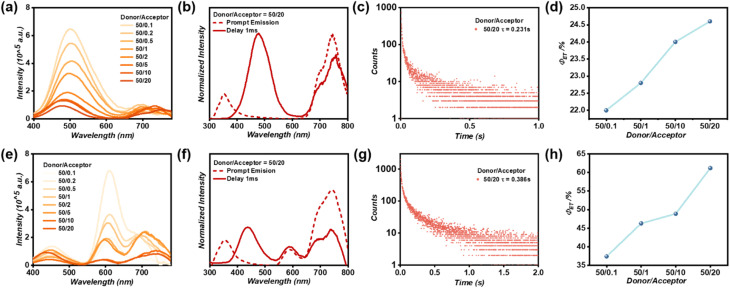
(a) Delayed spectra (duration = 1 ms) of SAPs@TLTP with the incorporation of different molar ratios of Cy5 under 280 nm excitation; (b) Normalized prompt (dashed line) and 1ms-delayed (solid line) emission spectra of SAPs@TLTP/Cy5 (the ratio of TLTP/Cy5 is 50/20) under 280 nm excitation; (c) Phosphorescence lifetime decay profile of SAPs@TLTP/Cy5 (the ratio of TLTP/Cy5 is 50/20) under 280 nm excitation at 753 nm; (d) *Φ*_ET_ at different SAPs@TLTP/Cy5 molar ratios; (e) delayed spectra (duration = 1 ms) of SAPs@TLTP/Cy3 with the incorporation of different molar ratios of Cy5 under 280 nm excitation; (f) normalized prompt (dashed line) and 1 ms delay (solid line) emission spectra of SAPs@TLTP/Cy3/Cy5 (the ratio of TLTP/Cy5 is 50/20) under 280 nm excitation; (g) phosphorescence lifetime decay profile of SAPs@TLTP/Cy3/Cy5 (the ratio of TLTP/Cy5 is 50/20) under 280 nm excitation at 753 nm; and (h) *Φ*_ET_ at different SAPs@TLTP/Cy3/Cy5 molar ratios.

Comparative studies with SAPs@TLTP/Cy3 revealed that despite the increased spectral overlap between the phosphorescent emission of SAPs@TLTP and the absorption profile of RB6G, the FRET efficacy is critically governed by noncovalent donor–acceptor interactions and dipole alignment orientations. Tunable persistent room-temperature phosphorescence (pRTP) emission, particularly in the near-infrared (NIR) region above 750 nm, remains a critical challenge for organic pRTP materials.^52^ In contrast, Förster resonance energy transfer between the singlet states of chromophores has been extensively used as an effective strategy to achieve NIR-delayed fluorescence in conventional fluorescent systems. Motivated by this principle, we subsequently implemented Cy5 coassembly with SAPs@TLTP, successfully achieving delayed fluorescence emission centered at 753 nm through energy transfer processes. After the SAPs@TLTP/Cy5 molar ratio (*λ*_ex_ = 280 nm) was increased, the delayed emission spectra reveal two key spectral changes (Fig. 6a): (i) a progressive increase in the Cy5 emission intensity at 700 nm with a concomitant redshift of its maximum and (ii) a simultaneous attenuation and blueshift of the SAPs@TLTP phosphorescence peak at 500 nm. Notably, the initial weak NIR emission at ≈750 nm (Fig. 6a) suggests potential triplet-to-singlet energy transfer or charge separation processes within the hybrid system. This systematic evolution conclusively validates the occurrence of FRET, and the observed Stokes shift reached 473 nm. As shown in Fig. 6b, highly overlapping prompt and delayed spectra were observed, revealing the FRET progress as a proof of supplement. TRFS revealed that SAPs@TLTP/Cy5 (TLTP : Cy5 molar ratio = 50 : 20) exhibited a delayed fluorescence lifetime of 0.231 s at 753 nm (Fig. 6c). Regrettably, the emission intensity of Cy5 following delayed sensitization did not significantly increase. Furthermore, analysis of the phosphorescence lifetime decay profiles 500 nm (Fig. S14) revealed a modest FRET efficiency (*Φ*_ET_ = 24.6%; Fig. 6d), attributable to the limited spectral overlap between the absorption of Cy5 and the phosphorescent emission of SAPs@TLTP. We also examined the optimal doping ratio for the SAPs@TLTP/Cy5 system. As depicted in Fig. S15, the near-infrared emission signal at around 700 nm becomes nearly undetectable when the donor/acceptor ratio increases from 50 : 20 to 50 : 60. Since the objective of this system is to achieve optimal near-infrared emission, it was determined that 50 : 20 is the optimal doping ratio for SAPs@TLTP/Cy5, based on a comprehensive evaluation of both emission intensity and wavelength.

To increase both the energy transfer efficiency and the NIR emission intensity, a cascaded FRET strategy was implemented. Building on the exceptional 618 nm afterglow from the SAPs@TLTP/Cy3 system, we designed a two-step energy transfer cascade with Cy5 as a secondary acceptor. This selection was guided by the strong spectral overlap between Cy3 emission and Cy5 absorption (Fig. S16). With increasing doping ratio between SAPs@TLTP/Cy3 and Cy5, the NIR emission peak progressively redshifted to 753 nm (Fig. 6e). Specifically, the newly emerged NIR emission increased significantly, accompanied by a marked reduction in the Cy3 emission intensity at 618 nm, confirming the occurrence of singlet–singlet Förster resonance energy transfer (SS-FRET) within this doped system (Fig. 6e). The high overlap of the prompt and delayed spectra in Fig. 6f also elucidates the two-step FRET progress. After Cy5 incorporation, the emission lifetime of the intermediate guest Cy3 at 618 nm decreased from 0.626 s to 0.484 s, achieving an SS-FRET efficiency of 22.6% (Fig. S17). TRFS analysis revealed ultralong emission lifetimes of 0.386 s at 753 nm at room temperature, attributing the persistent NIR luminescence to triplet excitation-driven singlet sensitization of Cy5 (Fig. 6g). In this system, the occurrence of NIR emission progresses through two energy transfers: (i) direct energy migration from the T_1_ excitons of SAPs@TLTP to S_1_ excitations of Cy5 and (ii) cascaded energy transfer *via* the intermediate Cy3 acceptor, where partial Cy3 excitations further transfer energy to Cy5. This cooperative pathway yielded a cascaded energy transfer efficiency of 61.2% (Fig. 6h), which was calculated from the phosphorescence lifetime decay profiles shown in Fig. S18. Additionally, we explored the optimal doping ratio for the ternary SAPs@TLTP/Cy3/Cy5 system. As illustrated in Fig. S19, the near-infrared emission at 700 nm is significantly quenched with increasing Cy5 concentration. Therefore, based on evaluation of both emission intensity and wavelength, a 50 : 20 ratio was selected as optimal.

**Fig. 7:**
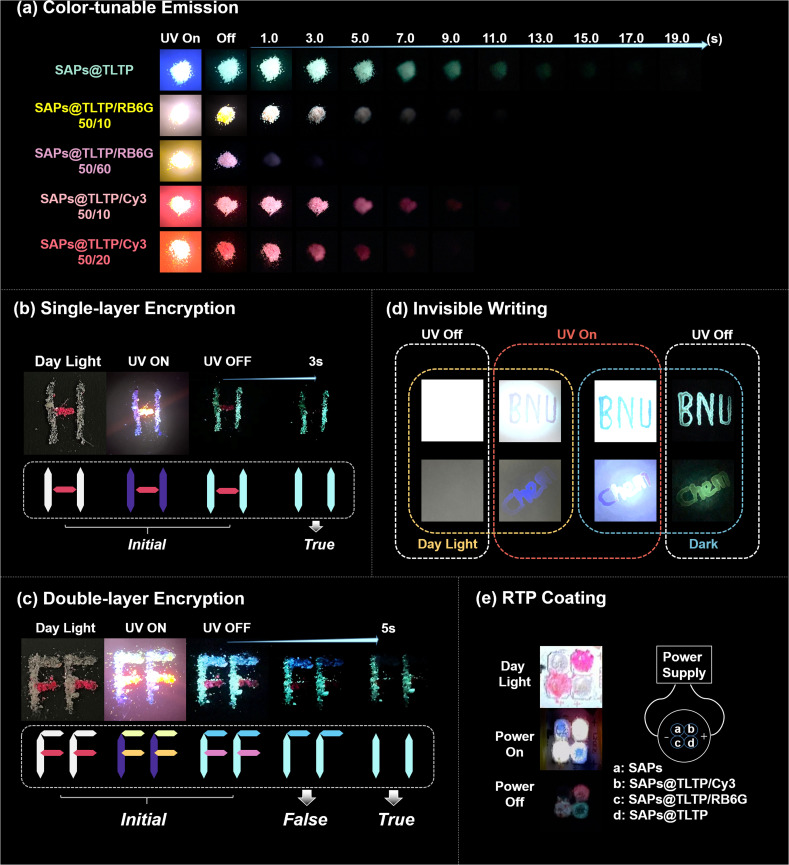
(a) Color-tunable and afterlow-tunable materials through FRET; (b) single-layer encryption of two RTP materials; (c) double-layer encryption of two RTP materials; (d) invisible writing by TLTP aqueous solution and university of lactose functionalization; and (e) RTP coating adhering to LEDs.

By adjusting the types and ratios of doped fluorescent materials, we achieved color-tunable, long-lived, and ultra-large Stokes shift organic RTP materials *via* a FRET strategy (Fig. 7a). These RTP materials, which exhibit different phosphorescence lifetimes, have the potential for use in encryption, information storage, and information security. As shown in Fig. 7b, we achieved single-layer optical encryption through the distinct lifetimes of the two materials, SAPs@TLTP and SAPs@TLTP/Cy3 (50/20). In pattern “H”, “I” was constructed with SAPs@TLTP, while “–” was constructed with SAPs@TLTP/Cy3 (50/20). Considering that the SAPs@TLTP has a longer afterglow than the SAPs@TLTP/Cy3 does (50/20), we anticipated the actual information to be the digit “11”. This assumption was indeed confirmed experimentally. Under daylight, the “I” appeared gray-white, and the “–” appeared pink. Under 280 nm UV light, they emitted corresponding fluorescence, blue-violet and bright yellow. After the UV light was turned off, the green “I” and pink “–” that formed the letter “H” remained observable. After 5 seconds, owing to the shorter lifetime of SAPs@TLTP/Cy3 (50/20), only the digit “11” formed by the green “I” remained visible. Furthermore, we introduced an additional RTP material, SAPs@TLTP/Cy3 (50/0.1), with a phosphorescence lifetime intermediate between that of SAPs@TLTP and SAPs@TLTP/Cy3 (50/20), to achieve two-layer encryption. As shown in Fig. 7c, the initial pattern “FF” was visible under both daylight and UV irradiation. However, an erroneous message appeared 2 seconds after the UV was turned off, and the genuine digital information “11” became visible only after 5 seconds.

Considering that lactose-functionalized TLTP has abundant hydrogen bonding sites, it is anticipated to be compatible with various matrices capable of providing effective hydrogen bonding. In this study, we selected readily available C18 reverse phase silica gel plates and filter paper. Both plates and filter paper exhibit low background fluorescence and moderate hygroscopicity. As shown in Fig. 7d, we wrote “BNU” and “Chem” *via* a 10 mg mL^−1^ aqueous solution of TLTP onto the C18 plate and filter paper, respectively. Owing to the excellent water solubility of TLTP, the patterns were not directly observable under daylight but became visible under UV irradiation. Visible afterglow remained observable even after the UV was turned off. Therefore, the lactose-functionalized TLTP is suitable for multiple hydrogen-bonding matrices, and its aqueous solution can also function as “invisible ink”, exhibiting excellent prospects for information encryption applications. Interestingly, we also confirmed the potential of these RTP materials for luminescent devices by adhering them as emitting coatings onto 280 nm UV LED beads (Fig. 7e). Under daylight, LED beads “a” and “d” appeared white, “b” appeared deep red, and “c” appeared light pink. Under UV irradiation, owing to the minimal distance between the phosphor and the light source, beads “a” and “d” appeared blue, whereas beads “b” and “c” appeared bright yellow. However, after the UV light was turned off for a period, beads “b”, “c”, and “d” exhibited pink, white, and green phosphorescence, respectively.

## Conclusions

We synthesized a TATP that does not exhibit room-temperature phosphorescence because of aggregation-induced quenching but displays intrinsic long-lived emission (*τ* = 1.53 s) at 77 K, confirming the phosphorescent capability of its TeP unit. To unlock this potential under ambient conditions, lactose functionalization was employed to harness intermolecular hydrogen bonding, effectively increasing TeP unit separations and suppressing aggregation. The resulting conjugate TLTP achieved RTP with a lifetime of 25.9 ms at room temperature. Further embedding TLTP within superabsorbent polymers, which provide rigid environment, enabled ultralong RTP (*τ* = 1.27 s). Theoretical calculations confirm that lactosedirected TeP chromophore isolations are critical for the activation of TeP phosphorescence. In addition to achieving efficient RTP, we engineered highperformance FRET systems *via* onestep and two-step coassembly routes between the RTP donor and fluorescent acceptors. These systems exhibit long-lived multicolor afterglow, tunable emission redshifts (584–753 nm), and large Stokes shifts (up to 473 nm), *via* the synergy between RTP and FRET. Furthermore, the developed materials enabled advanced single/double-layer information encryption and revealed excellent functionality in prototype optical devices. This work represents the first discovery of lactose's role as a molecular “bridge” connecting novel hydrophilic uncrystallized phosphorescent materials with their known hydrophobic crystallized counterparts, which not only reveals the untapped RTP potential of TeP-based chromophores but also establishes lactose mediated supramolecular control as a versatile platform for designing stimuli-responsive phosphorescent materials.

## Author contributions

Conceptualization, X. Niu and G.-W. Xing; methodology, X. Niu; validation, X. Niu, M.-X. Chen and Y. Zhang; formal analysis, X. Niu, M.-X. Chen and Y. Zhang; investigation, X. Niu, M.-X. Chen, Y. Zhang, G.-J. Liu, S.-H. Qian, H. Zhang and J.-Q. Jiang; visualization, X. Niu; writing – original draft, X. Niu; writing – review & editing, X. Niu, M.-X. Chen, H.-W. Tan and G.-W. Xing; funding acquisition, H.-W. Tan and G.-W. Xing; resources, H.-W. Tan and G.-W. Xing; supervision, H.-W. Tan and G.-W. Xing.

## Conflicts of interest

The authors declare that they have no conflicts of interest.

## Supplementary Material

SC-OLF-D6SC01359G-s001

## Data Availability

The data supporting this article have been included as part of the supplementary information (SI). Supplementary information is available. See DOI: https://doi.org/10.1039/d6sc01359g.
