# Supplementary material for: Ultralong organic room-temperature phosphorescence of tetraphenylene via lactose functionalization and chromophore isolation
Source: Chem Sci. 2026 Apr 13;17(23):11368–78. doi: 10.1039/d6sc01359g (PMC13159015; doi:10.1039/d6sc01359g)
Supplement: SC-017-D6SC01359G-s001 [file SC-017-D6SC01359G-s001.pdf]

Supplementary Information (SI) for Chemical Science.
This journal is © The Royal Society of Chemistry 2026

Supporting Information

Ultralong Organic Room-Temperature Phosphorescence of Tetraphenylene via Lactose Functionalization and Chromophore Isolation

Xin Niu,[a]‡ Mingxing Chen[b]‡ Yuan Zhang,[a]‡ Guang-jian Liu,[a] Su-Hang Qian,[a] Hui Zhang,[a] Jia-Qing Jiang,[a] Hongwei Tan* [a] and Guo-wen Xing*[a]

[a] College of Chemistry, Beijing Normal University Beijing 100875, China

[b] College of Chemistry and Molecular Engineering, Peking University, Beijing 100871, China

E-mail: gwxing@bnu.edu.cn; hongwei.tan@bnu.edu.cn

‡ X. Niu, M.X. Chen and Y. Zhang contributed equally to this work.

Content

1. General information 1

2. Synthesis of TATP and TLTP...2

3. RDG of TATP and TLTP..5

4. Vibrational analysis of TLTP...5

5. RMSD analysis of TLTP and TATP..6

6. Preparation and PL Properties of SAPs@TLTP...6

7. Preparation and PL Properties of SAPs@TLTP/dye (Cy3, Cy5, RB6G) 8

8. NMR spectra and HRMS ……...12

9. HPLC of TLTP ……...17

10. Cartesian coordinates and corresponding energies for DFT Calculation…………..........................18

11. Reference 35

1. General information

Materials

Materials and reagents were purchased from commercial suppliers and used without further purification unless otherwise stated. The Super Absorbent Polymer (SAP) is a typical functional polymeric material, primarily composed of sodium polyacrylate with a low degree of cross-linking, containing strong hydrophilic groups. The SAP used in this work was sourced from Shanghai Yuanye Bio-Technology Co., Ltd (Product Code: S29884-500g). All solvents were analytical pure unless otherwise noted. Double-distilled water was used throughout all the experiments. All anaerobic reactions were performed under a dry N_2 atmosphere. All reactions were monitored by thin-layer chromatography (TLC) on T-HSGF10025025 normal-phase silica gel glass plates or 60 RP-18 F254s reversed-phase silica gel glass plates and revealed with UV light (254 nm or 365 nm) or EtOH-H_2SO_4 (7%) solution. Flash column chromatography was performed on 200-300 mesh silica gel. Reversed-phase column was performed on SiliaSphere C18 (50 μm, 120 Å). Molecular exclusion chromatography was performed on Bio-Gel® P-2 Media (45-90 μm).

Characterization

^{1}H and ^{13}C NMR spectra were recorded on JNM-ECZR spectrometer (400 and 600 MHz) with tetramethyl silane (TMS) as an internal standard. The residual peaks of the solvent were chloroform-d1 at 7.26 ppm (^{1}H) and 77.2 ppm (^{13}C), and DMSO-d_6 at 2.50 ppm (^{1}H) and 39.5 ppm (^{13}C). High resolution mass spectra were recorded on a Bruker MICROTOF-QII mass spectrometer (EI) or Bruker autoflex speed LRF (MALDI-TOF). Photoluminescence (PL) spectral tests including fluorescence and phosphorescence spectra and time-resolved decay of all the samples were collected on an Edinburgh FLS980 fluorescence spectrometer. The solid-state UV-Vis spectra were carried out on a UV3600PLUS spectrophotometer. The UV lamp used to take photos for fluorescent photos is 20-30mW.

Theoretical calculation

The optimization of TATP and TLTP was carried out by DFT calculation at B3LYP/6-31G(d) level with Grimme's D3 dispersion correction. The calculation of excited state energies was carried out by time-dependent DFT (TD-DFT) at the same computation level. All the DFT calculations were performed using Gaussian09 (Revision D.01) package.[1-4] Molecular Dynamics (MD) simulations were performed using the Tinker package (version 8.11.3).[5] The MM3.PRM all-atom force field[6] was used for the dimers of TATP and TLTP. The Tinker parameter file was employed without modification. Vibrational frequency analysis[7,8] was performed using the Gaussian 09

(Revision D.01) software package. The root-mean-square deviation (RMSD) was obtained by analyzing and comparing the molecular structures optimized with this software, utilizing the VMD program[9].

2. Synthesis of TATP and TLTP

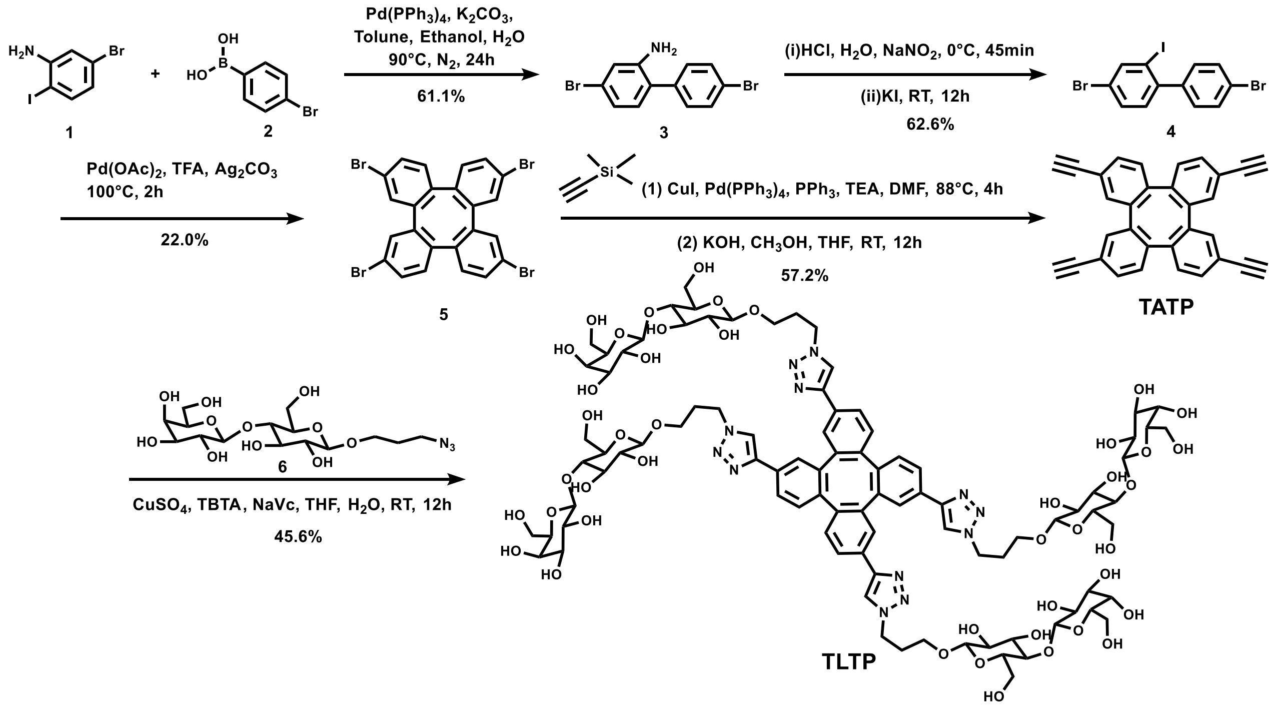

Scheme S1. Synthetic procedure and chemical structure of TATP and TLTP.

Compound **6** is synthesized according to the reported procedure.[10]

Compound **3**, **4** and **5** are synthesized according to the reported procedures.[11]

Synthesis of compound 3：To a 250 mL three-necked flask (equipped with ground-glass joints) were added a PTFE-coated magnetic stir bar, compound **1** (4.50 g, 15.0 mmol), compound **2** (4.00 g, 20.0 mmol), $Pd(PPh_3)_4$ (173 mg, 150 μmol) and K_2CO_3 (6.20 g, 45.0 mmol). The reaction flask was evacuated and back-filled with N_2, followed by the addition of toluene (27.0 mL), ethanol (18.0 mL) and double-distilled water (18.0 mL) with a gas-tight syringe. The reaction mixture was stirred and heated to 90 °C . After the reaction mixture was stirred at 90 °C for 24h, it was allowed to cool to room temperature. The reaction mixture was extracted with dichloromethane (DCM), and the combined organic phases were washed with double-distilled water. The organic phases were dried over anhydrous $MgSO_4$, and concentrated in vacuo. The crude product was purified by silica gel column chromatography with petroleum ether (PE) / DCM (4:1, v/v) to give the corresponding compound **3** (3.00 g, 61.1%).

1H NMR (600 MHz, Chloroform-d) δ (ppm): 7.57 (d, J = 8.5 Hz, 2H), 7.29 (d, J = 8.5 Hz, 2H), 6.93 (s, 2H), 6.92 (s, 1H), 3.87 (s, 2H).

Synthesis of compound 4: To a 100 mL round-bottom flask (equipped with ground-glass joints) were added a PTFE-coated magnetic stir bar, HCl (36-38%; 11.6 mmol, 1.0 mL) and double-distilled water (5.0 mL). Compound **3** (1.60 g, 5.00 mmol) was added slowly to the stirred mixture. An aqueous solution of $NaNO_2$ (690 mg, 10.0 mmol) in double-distilled water (2.0 mL) was added and the reaction mixture was stirred in ice water bath for 1h. An aqueous solution of KI (2.10 g 12.5 mmol) in double-distilled water (2.0 mL) was added and the reaction mixture was stirred at room temperature for 12h. The mixture after reaction was washed successively with 1M HCl, saturated sodium bicarbonate solution, saturated sodium thiosulfate solution and saturated sodium chloride solution. Then extracted with DCM, and the combined organic extracts were dried over anhydrous $MgSO_4$, and concentrated in vacuo. The crude product was purified by silica gel column chromatography with PE to give the corresponding compound **4** (1.34 g, 62.6%).

1H NMR (600 MHz, Chloroform-d) δ (ppm): 8.10 (s, 1H), 7.56 (d, J = 8.5 Hz, 2H), 7.52 (d, J = 8.2 Hz, 1H), 7.18 (d, J = 8.5 Hz, 2H), 7.13 (d, J = 8.2 Hz, 1H).

Synthesis of compound 5: A 35 mL thick-walled tube (with a Teflon high pressure valve) equipped with a magnetic stir bar was charged with $Pd(OAc)_2$ (10.1 mg, 45.0 μmol) followed by compound **4** (131 mg, 300 μmol), Ag_2CO_3 (41.4 mg, 150 μmol) and TFA (2.5 mL). The tube was placed into an oil bath preheated to 100 °C . After the reaction mixture was stirred at 100 °C for 2 h, it was allowed to cool to room temperature. The reaction mixture was diluted with DCM and then filtered through a small pad of Celite. The filtrate was neutralized with saturated aqueous $NaHCO_3$. The aqueous phase was separated and back-extracted with DCM. The combined organic phases were dried over anhydrous $MgSO_4$ and concentrated in vacuo. The crude product was purified by silica gel column chromatography with PE to give the corresponding compound **5** (20.0 mg, 22.0%).

1H NMR (600 MHz, Chloroform-d) δ (ppm): 7.45 (d, J = 8.2 Hz, 4H), 7.31 (s, 4H), 7.01 (d, J = 8.3 Hz, 4H).

Synthesis of compound TATP: (1) A 35 mL thick-walled tube (with a Teflon high pressure valve) equipped with a magnetic stir bar was charged with $Pd(PPh_3)_4$ (18.6 mg, 16.1 μmol), followed by compound **5** (100 mg, 161 μmol), PPh_3 (4.20 mg, 16.1 μmol) and CuI (2.50 mg, 12.9 μmol). The reaction tube was evacuated and back-filled with N_2, followed by the addition of Triethylamine (TEA, 1.0 mL), *N*,*N*-Dimethylformamide (DMF, 1.0 mL) and trimethylsilylacetylene (0.1 mL, 726 μmol). The tube was placed into an oil bath preheated to 88 °C . After the reaction mixture was stirred at 88 °C for 4 h, it was allowed to cool to room temperature. The reaction mixture was diluted with DCM and then filtered through a small pad of Celite. The filtrate was neutralized with double-distilled water. The aqueous phase was separated and back-extracted with DCM. The combined organic

phases were dried over anhydrous $MgSO_4$ and concentrated in vacuo. There was no need to purify the crude product after workup in this step and the crude product can be directly used in the next step. (2) To a 25 mL round-bottom flask (equipped with ground-glass joints) were added a PTFE-coated magnetic stir bar, the entire crude product from the previous step, and tetrahydrofuran (THF, 5.0 mL). An aqueous solution of KOH (65.2 mg, 1.16 mmol) in CH_3OH (5.0 mL) was added and the reaction mixture was stirred at room temperature overnight. The reaction mixture was neutralized with 1M HCl. Then the mixture was washed successively with saturated sodium bicarbonate solution and saturated sodium chloride solution. The aqueous phase was separated and back-extracted with DCM. The combined organic phases were dried over anhydrous $MgSO_4$ and concentrated in vacuo. The crude product was purified by silica gel column chromatography with PE/DCM (40/1, v/v) to give the corresponding compound **TATP** (37.0 mg, 57.2%).

^{1}H NMR (600 MHz, Chloroform-d) δ (ppm): 7.43 (d, J = 7.9 Hz, 4H), 7.31 (s, 4H), 7.11 (d, J = 7.9 Hz, 4H), 3.07 (s, 4H). ^{13}C NMR (151 MHz, Chloroform-d) δ (ppm): 141.16, 140.45, 132.88, 131.55, 129.22, 121.82, 83.03, 78.16. HRMS (EI): m/z calcd for $[C_{32}H_{16}]^+$: 400.1247; Found: 400.1249.

Synthesis of compound TLTP: To a 25 mL round-bottom flask (equipped with ground-glass joints) were added a PTFE-coated magnetic stir bar, compound **TATP** (20.0 mg, 49.9 μmol)，compound **6** (216 mg, 300 μmol) and THF (4.0 mL). Tris(benzyltriazolylmethyl)amine (TBTA, 10.6 mg, 20.0 μmol) was mixed with the aqueous solution of $CuSO_4$ (3.20 mg, 20.0 μmol) in double-distilled water (1.0 mL), dispersed by ultrasound, then an aqueous solution of sodium ascorbate (NaVc, 7.90 mg, 40.0 μmol) in double-distilled water was added. The above mixture was added to the reaction system solution and the reaction mixture was stirred at room temperature for 12h. The following process was to remove the solvent by a rotary evaporator, the crude product was purified by size exclusion chromatography (P2) with H_2O to give the corresponding compound **TLTP** (47.8 mg, 45.6%).

^{1}H NMR (600 MHz, DMSO-d$_6$) δ (ppm): 8.65 (m, 4H), 7.87 (d, J = 7.4 Hz, 4H), 7.72 (s, 4H), 7.34 (d, J = 7.4 Hz, 4H), 5.23 (s, 2H), 5.11 (s, 2H), 4.95 (s, 1H), 4.81 (s, 2H), 4.67 (m, 8H), 4.54 (m, 8H), 4.50 (s, 8H), 4.20 (m, 6H), 3.79 (s, 2H), 3.73 (s, 2H), 3.66 (s, 2H), 3.60 (m, 12H), 3.53 (s, 4H), 3.48 (m, 12H), 3.38 (s, 8H), 3.32 (s, 11H), 3.29 (m, 8H), 3.04 (s, 2H), 2.11 (s, 8H). ^{13}C NMR (101 MHz, DMSO-d$_6$) δ (ppm): 145.65, 141.35, 140.02, 130.40, 129.81, 125.79, 124.56, 122.03, 103.90, 102.57, 80.73, 75.55, 74.91, 74.84, 73.26, 71.72, 71.64, 70.78, 70.58, 68.19, 65.50, 60.44, 46.73, 30.02. MALDI-TOF MS : m/z calcd for $[C_{92}H_{124}N_{12}O_{44}]^+$: 2100.78; Found: 2100.61.

3. RDG of TLTP and TATP

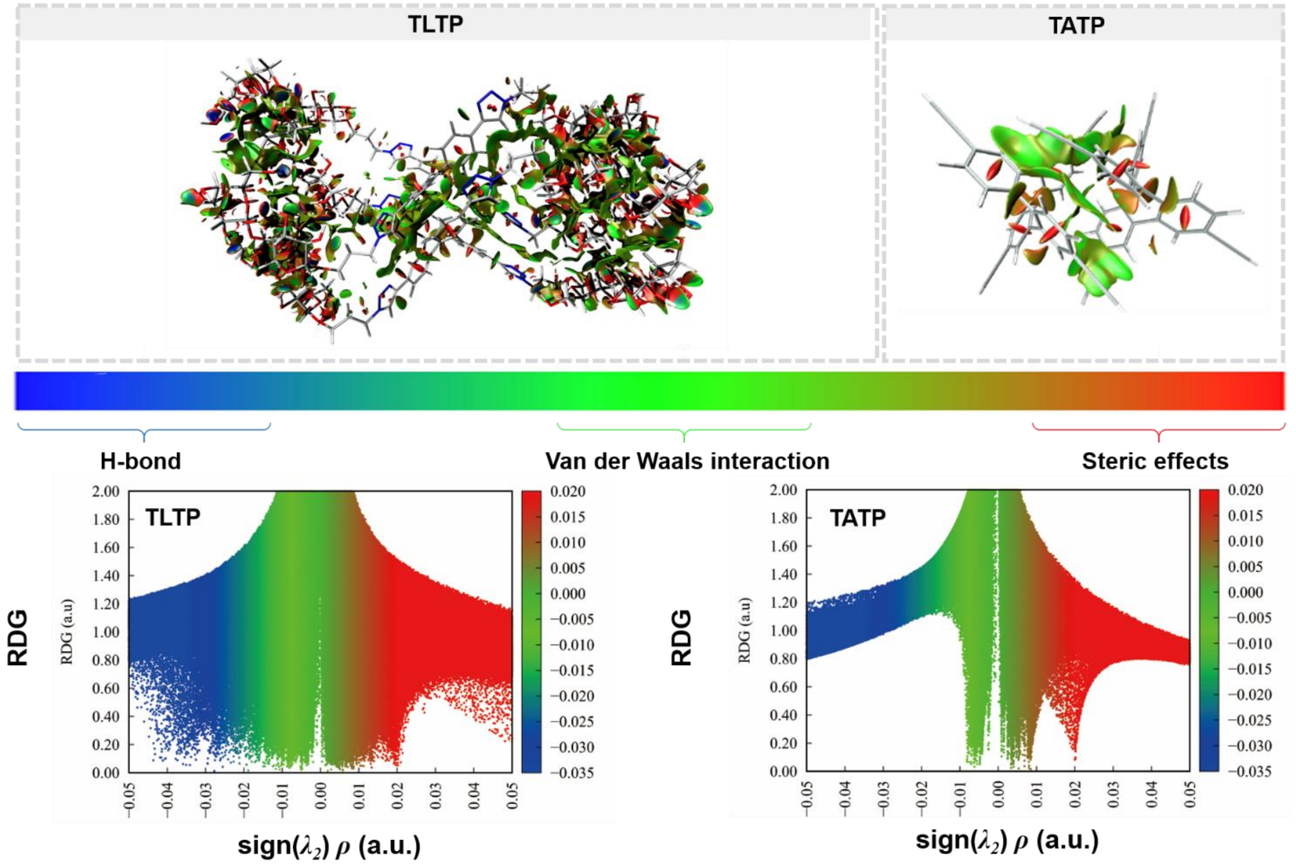

Figure S1. RDG isosurfaces of TLTP and TATP with an RDG of 0.5 are colored on the basis of the sign of λ2 and the function of the reduced density gradient and d sign (λ2) ρ scatter spectra for TLTP and TATP.

4. Vibrational analysis of TLTP

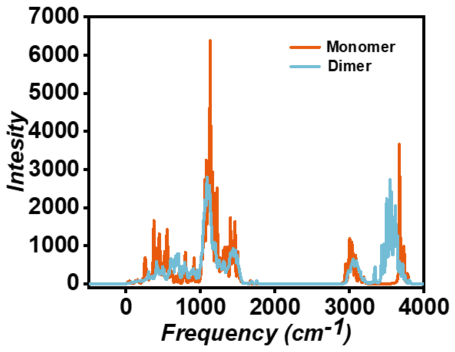

Figure S2. Vibrational analysis spectra of TLTP monomer and TLTP dimer

5. RMSD analysis of TLTP and TATP

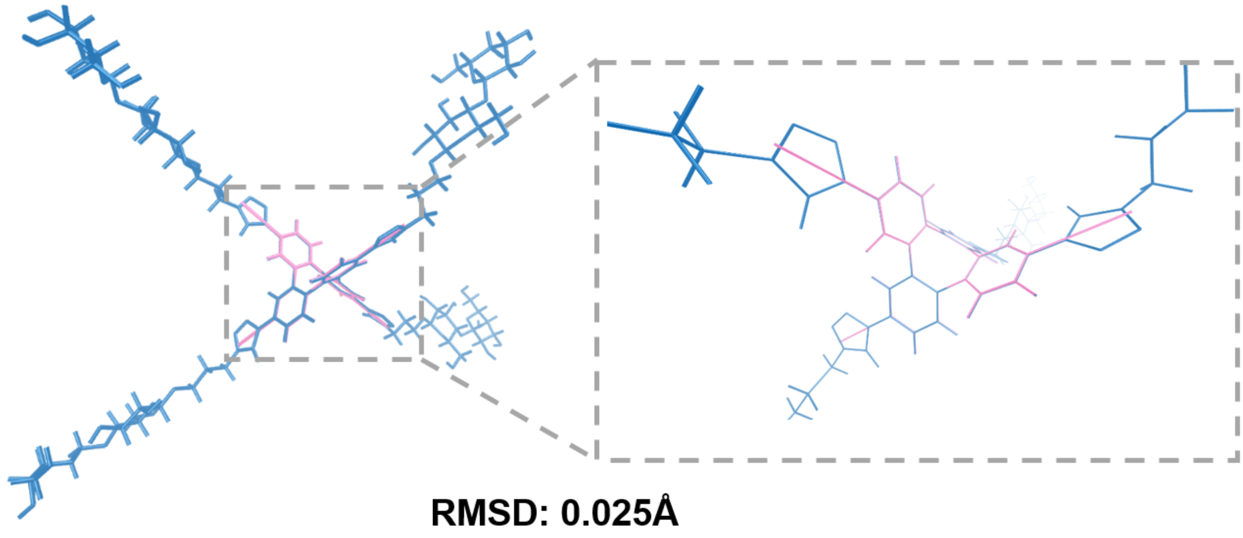

Figure S3. Visual representation of chromophore overlap between TLTP and TATP.

6. Preparation and PL Properties of SAPs@TLTP

Preparation of SAPs@TLTP

1 mL aqueous solution of TLTP (1 mg/mL) was added to 100 mg of SAPs (80-100 mesh). The mixture was allowed to stand for 12 hours, followed by drying in an oven at 110 °C for 6 hours. After cooling to room temperature, the resulting material was ground to yield SAPs@TLTP.

PL Properties of SAPs@TLTP

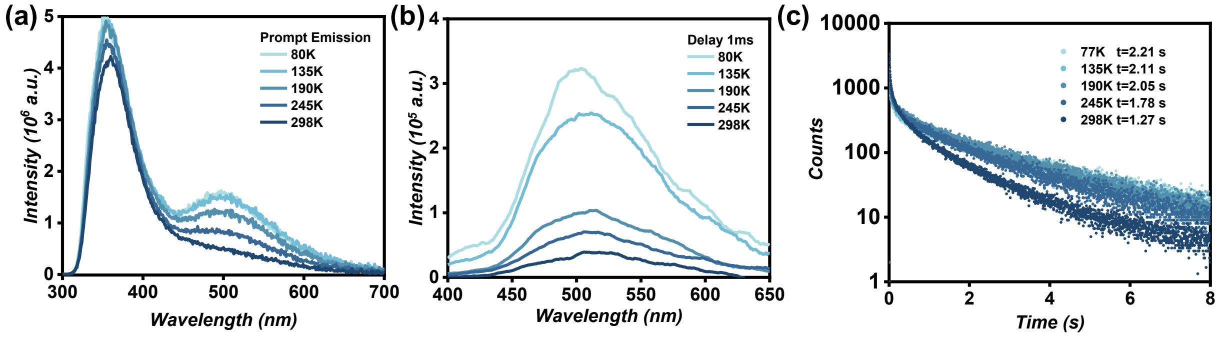

Figure S4. (a) Variable-temperature prompt emssion spectra of SAPs@TLTP under 280 nm excitation; (b) variable-temperature 1ms-delayed spectra of SAPs@TLTP under 280 nm excitation; (c) variable-temperature phosphorescence lifetime decay profiles of SAPs@TLTP were monitored at 500 nm under 280nm excitation.

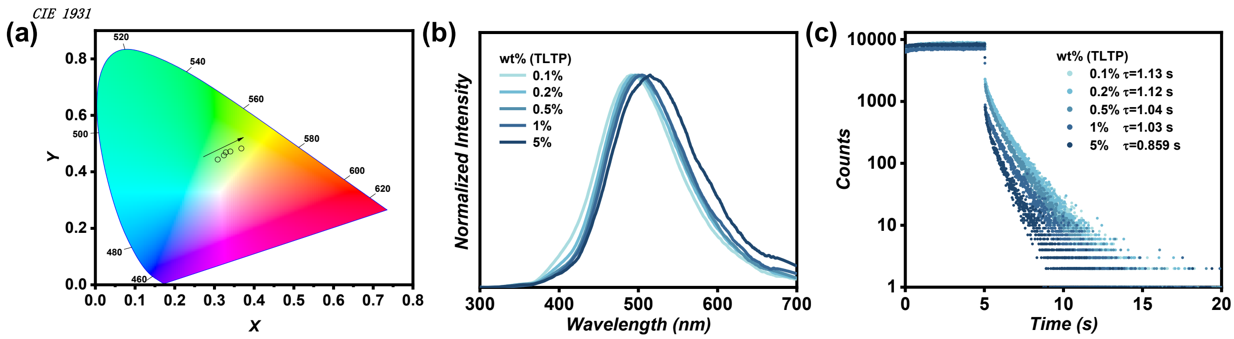

Figure S5. (a) CIE 1931 diagram showing the color shift in emission for SAPs@TLTP with different mass fraction ratios of TLTP; (b) delayed spectra (duration = 1 ms) of SAPs@TLTP with different mass fraction ratios of TLTP under 280 nm excitation; (c) phosphorescence lifetime decay profiles (measured at 500 nm) of SAPs@TLTP with different mass fraction ratios of TLTP under 280 nm excitation

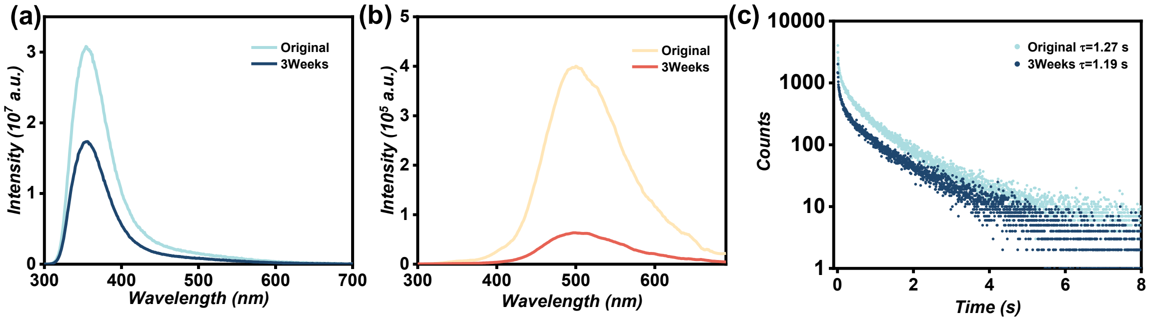

Figure S6. (a) Prompt emssion spectra of SAPs@TLTP at initial preparation and after three weeks of storage under 280 nm excitation; (b) delayed spectra (duration = 1 ms) of SAPs@TLTP at initial preparation and after three weeks of storage under 280 nm excitation; (c) phosphorescence lifetime decay profiles of SAPs@TLTP under 280nm excitation at initial preparation and after three weeks of storage were monitored at 500 nm under 280nm excitation.

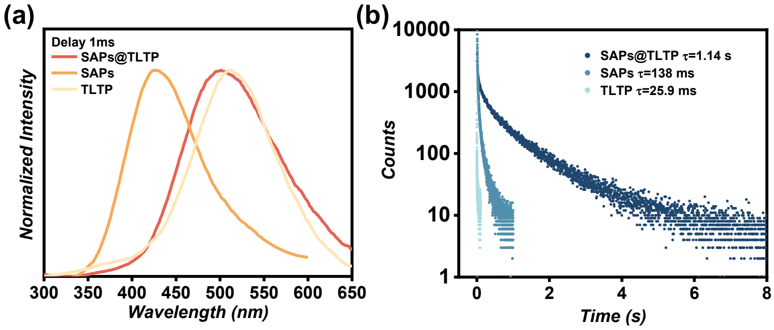

Figure S7. (a) Delayed spectra (duration = 1 ms) of SAPs, TLTP and SAPS@TLTP under 280 nm excitation; (b) phosphorescence lifetime decay profiles of SAPs (monitored at 425 nm), TLTP (monitored at 500 nm) and SAPS@TLTP (monitored at 500 nm) under 280nm excitation.

7. Preparation and PL Properties of SAPs@TLTP/dye (Cy3, Cy5, RB6G)

Preparation of SAPs@TLTP/dye (Cy3, Cy5, RB6G)

Aqueous solutions (1 mL each) containing TLTP (1 mg) and the corresponding ratios of Cy3, Cy5, or RB6G were prepared. Each solution was separately added to 100 mg of SAPs (80-100 mesh). The mixtures were allowed to stand for 12 hours, followed by drying in an oven at 110 °C for 6 hours. After cooling to room temperature, the resulting materials were ground to obtain the corresponding SAPs@TLTP/Cy3, SAPs@TLTP/Cy5, and SAPs@TLTP/RB6G composites.

PL Properties of SAPs@TLTP/dye (Cy3, Cy5, RB6G)

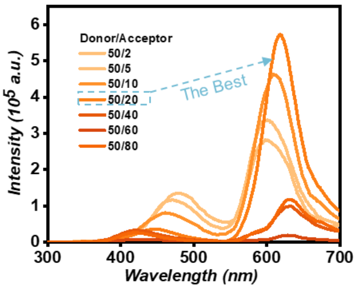

Figure S8. Delayed spectra (duration=1ms) of SAPs@TLTP with different molar ratios of Cy3 under 280 nm excitation.

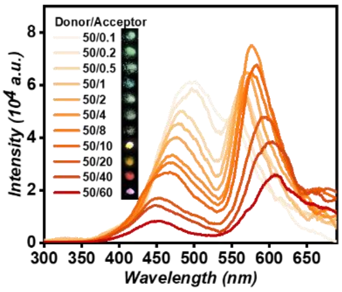

Figure S9. Delayed spectra (duration=1ms) of SAPs@TLTP with the incorporation of different molar ratios of RB6G (insert: photograph under 280 nm-UV excitation showing emission color change of SAPs@TLTP itself and with different molar ratios of RB6G) under 280 nm excitation.

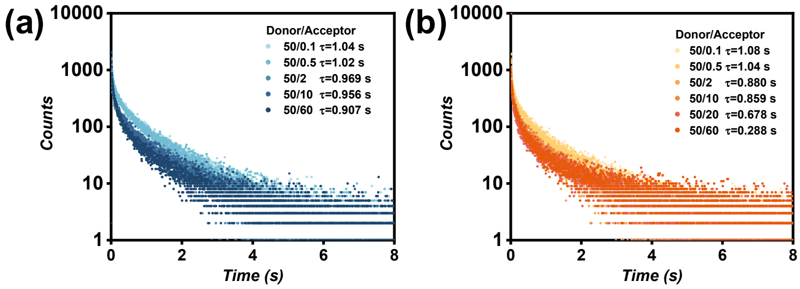

Figure S10. (a) Phosphorescence lifetime decay profiles (measured at 500 nm) of SAPs@TLTP with the incorporation of different molar

ratios of RB6G under 280 nm excitation; (b) phosphorescence lifetime decay profiles (measured at 580 nm) of SAPs@TLTP with the incorporation of different molar ratios of RB6G under 280 nm excitation.

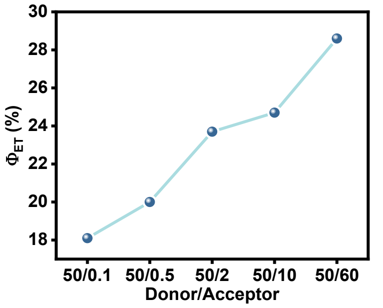

Figure S11. Φ_{ET} at different SAPs@TLTP/RB6G molar ratios.

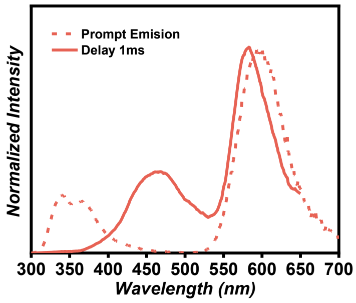

Figure S12. Normalized prompt (dashed line) and 1ms-delayed (solid line) emission spectra of SAPs@TLTP/RB6G under 280 nm excitation.

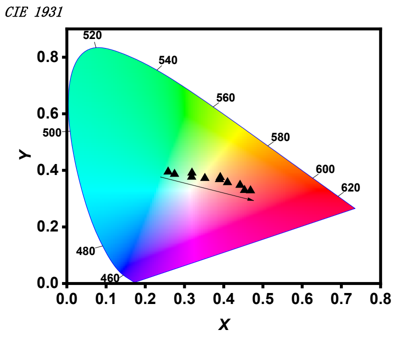

Figure S13. CIE 1931 diagram showing the color shift in emission for SAPs@TLTP with different mass fraction ratios of RB6G.

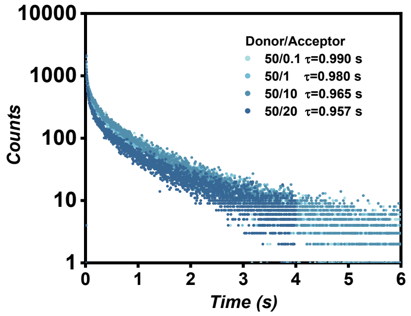

Figure S14. Phosphorescence lifetime decay profiles (measured at 500 nm) of SAPs@TLTP with the incorporation of different molar ratios of Cy5 under 280 nm excitation.

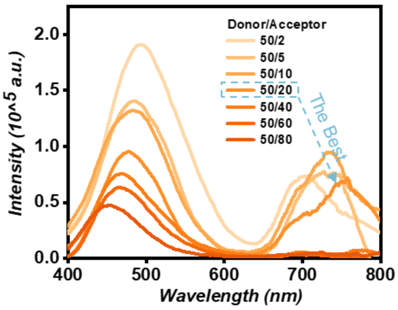

Figure S15. Delayed spectra (duration=1ms) of SAPs@TLTP with different molar ratios of Cy5 under 280 nm excitation.

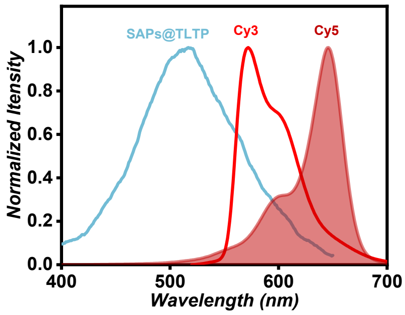

Figure S16. Normalized phosphorescence spectra of SAPs@TLTP and PL spectra Cy3 and absorption spectra Cy5 under 280 nm excitation and 365nm excitation.

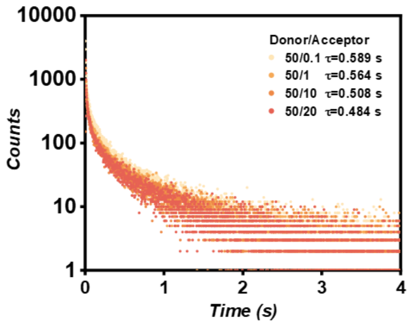

Figure S17. Phosphorescence lifetime decay at 618nm of SAPs@TLTP/Cy3 with the incorporation of different molar ratios of Cy5 under 280 nm excitation.

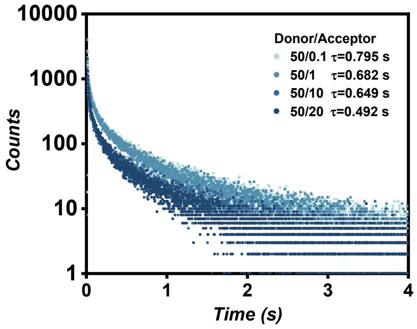

Figure S18. Phosphorescence lifetime decay profiles (measured at 450 nm) of SAPs@TLTP/Cy3 with the incorporation of different molar ratios of Cy5 under 280 nm excitation.

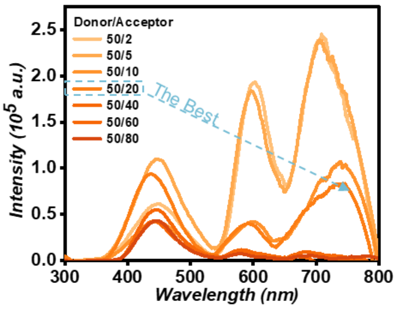

Figure S19. Delayed spectra (duration=1ms) of SAPs@TLTP/Cy3 with different molar ratios of Cy5 under 280 nm excitation.

8. NMR spectra and HRMS

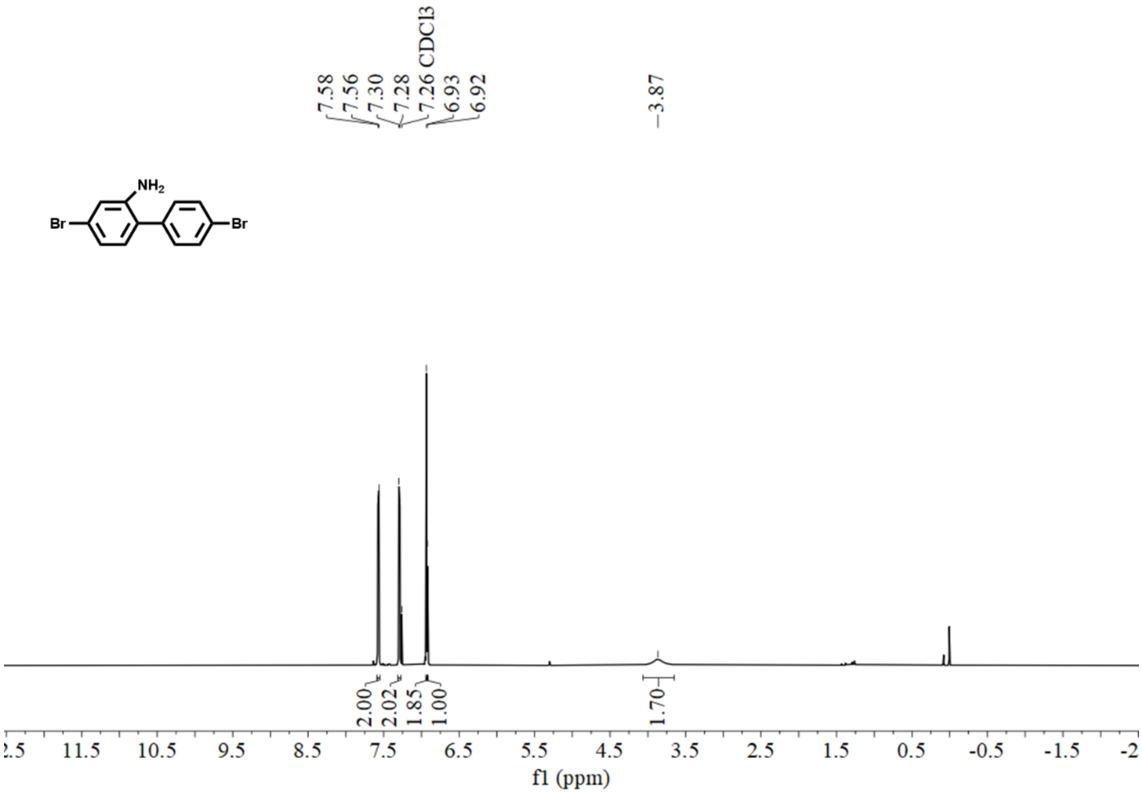

Figure S20. ^{1}H NMR spectra of compound **3** in $CDCl_3$.

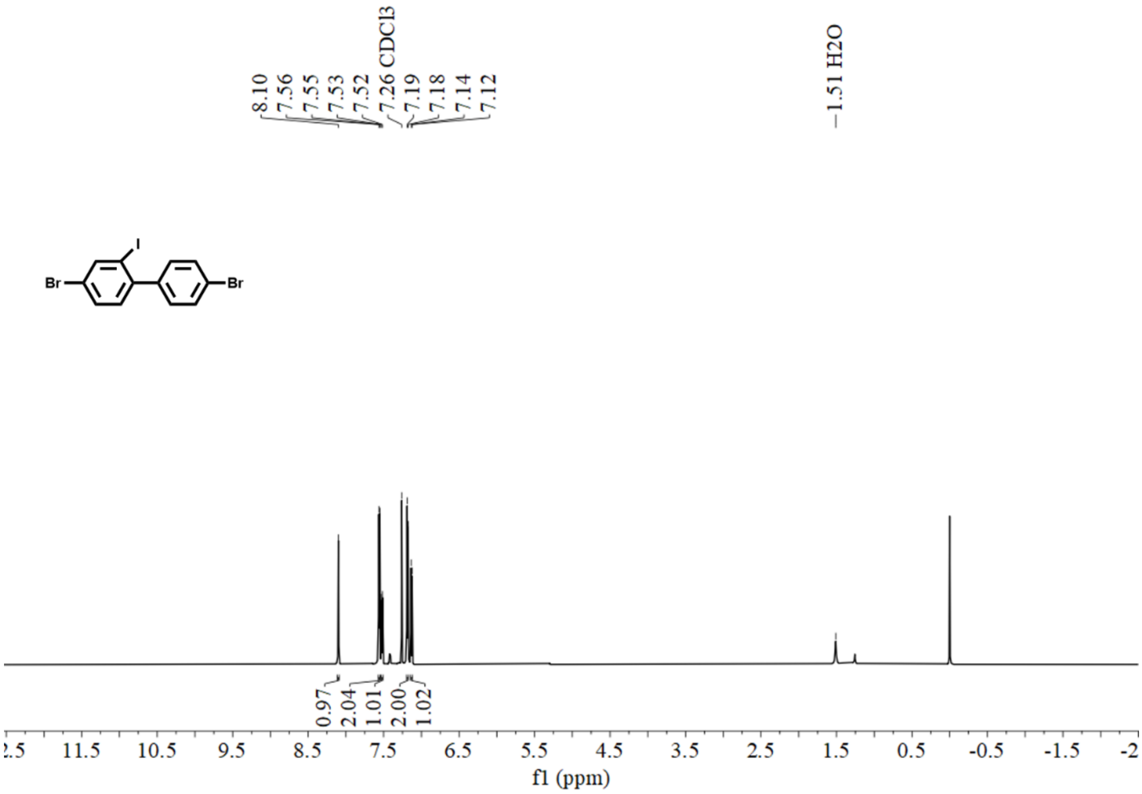

Figure S21. ^{1}H NMR spectra of compound **4** in $CDCl_3$.

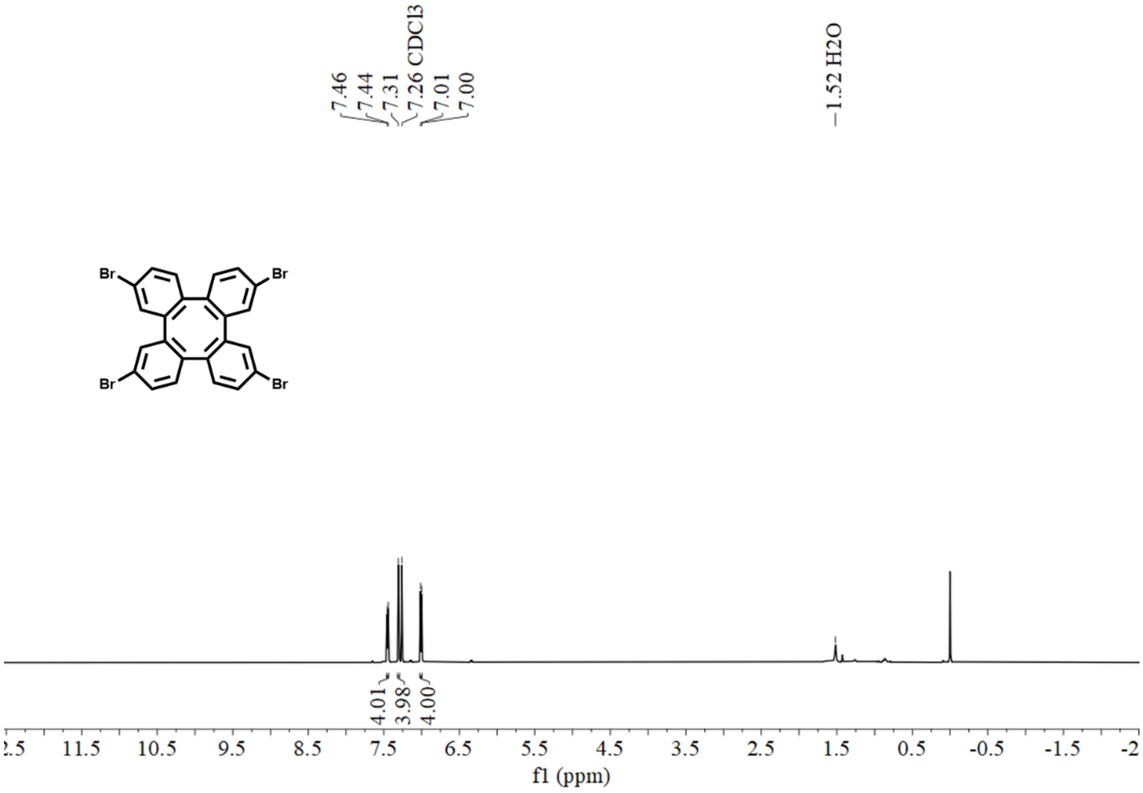

Figure S22. ^{1}H NMR spectra of compound 5 in $CDCl_3$.

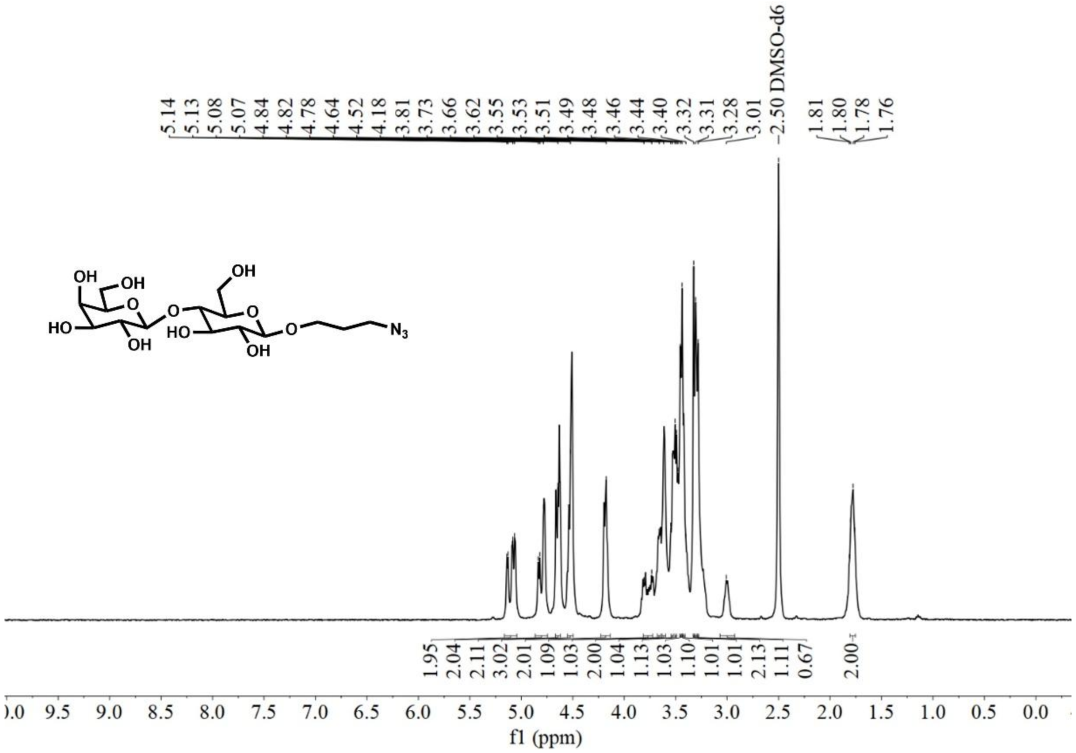

Figure S23. ^{1}H NMR spectra of compound **6** in DMSO-d_6.

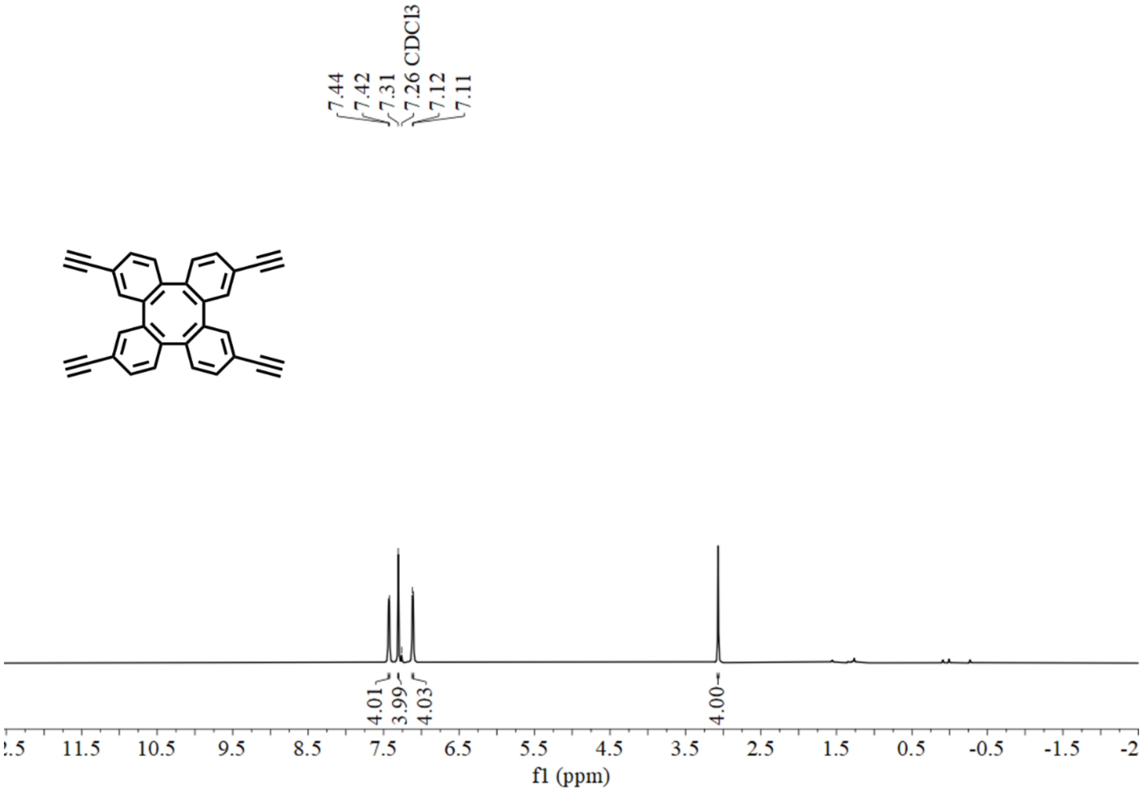

Figure S24. ^{1}H NMR spectra of compound **TATP** in $CDCl_3$.

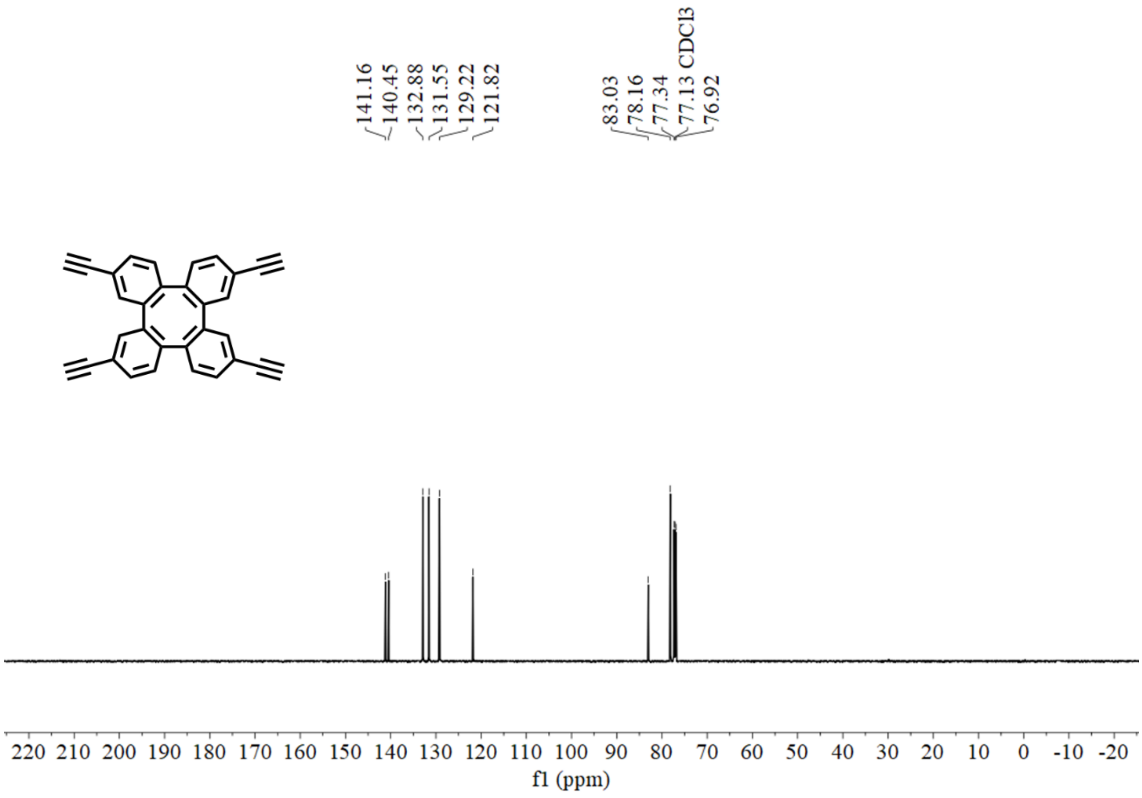

Figure S25. ^{13}C NMR spectra of compound **TATP** in $CDCl_3$.

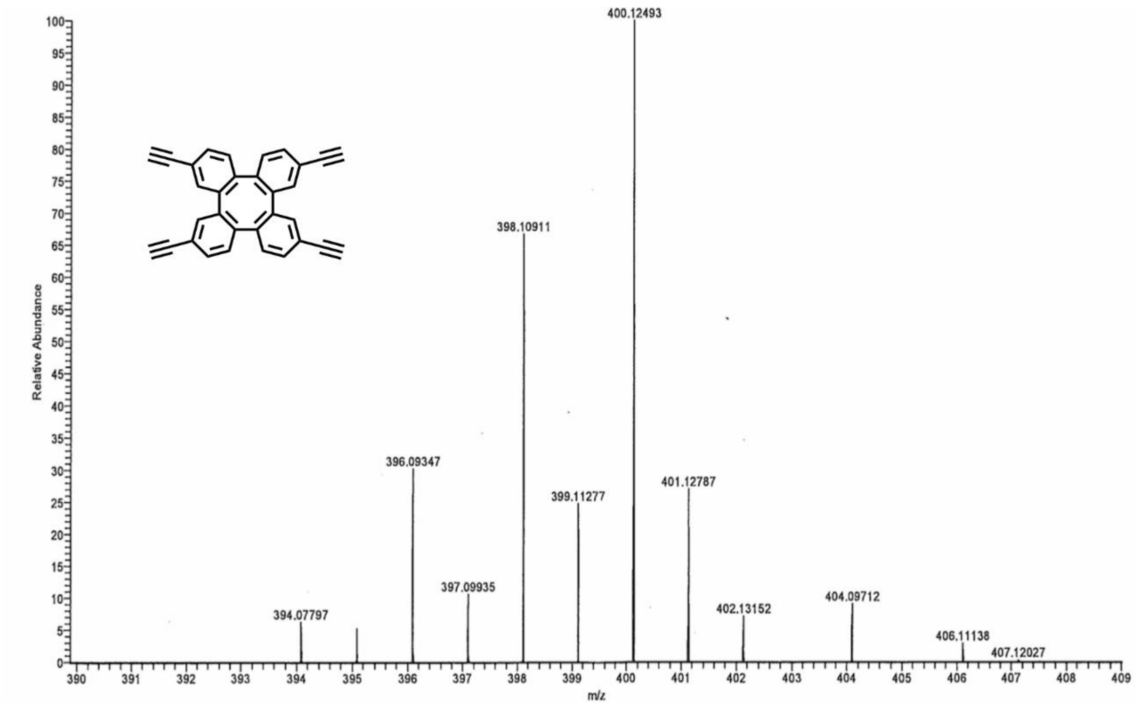

Figure S26. HRMS of Compound **TATP**.

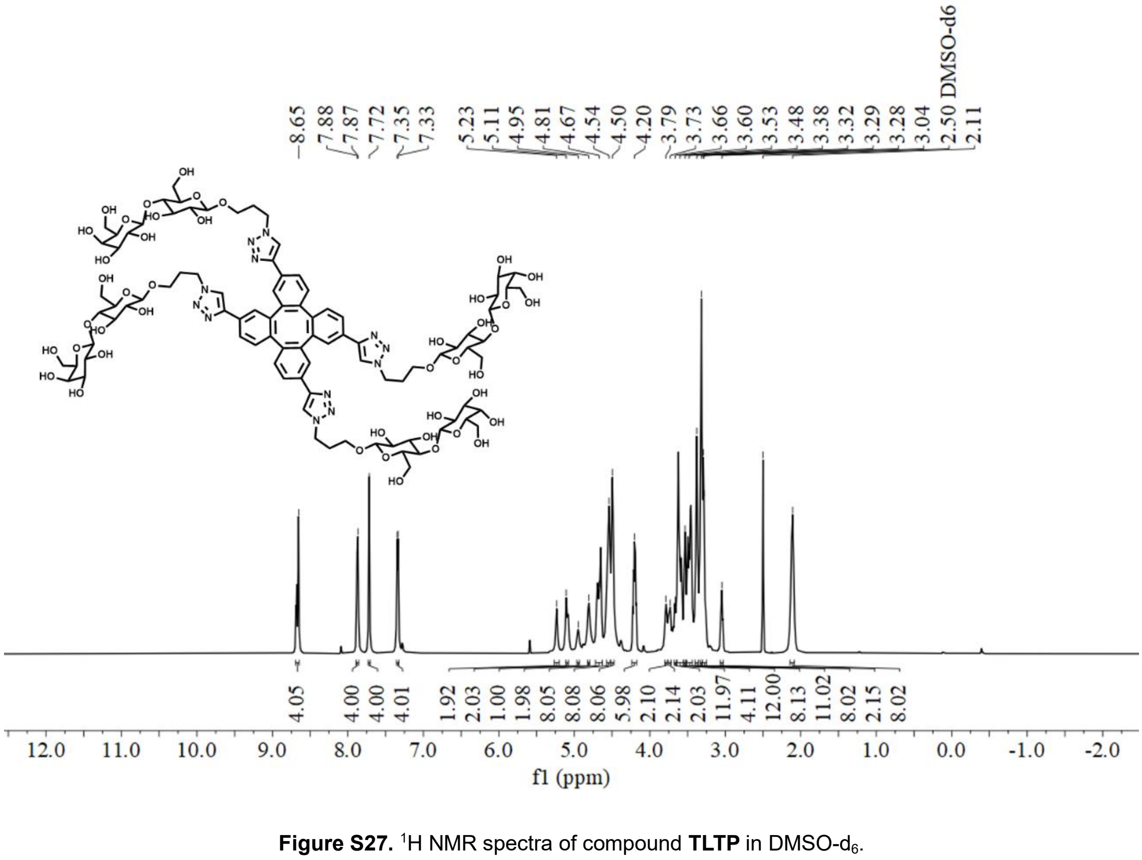

Figure S27. ^{1}H NMR spectra of compound **TLTP** in DMSO-d_6.

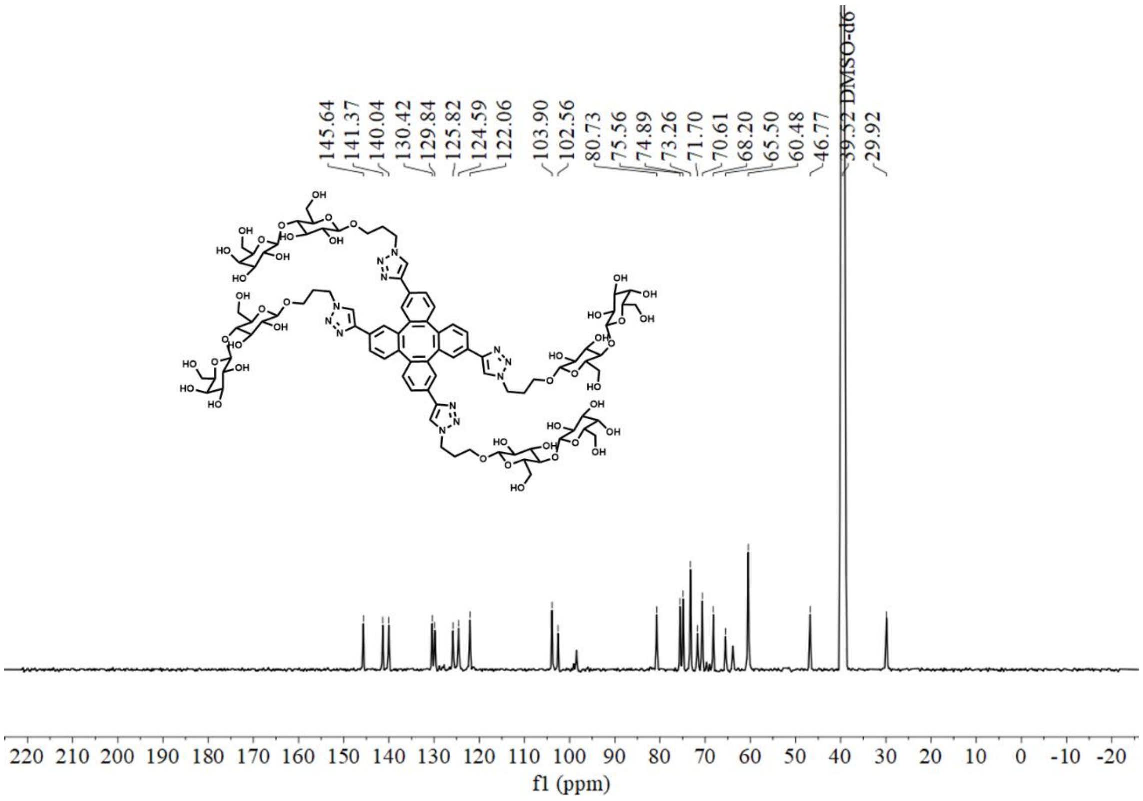

Figure S28. ^{13}C NMR spectra of compound **TLTP** in DMSO-d_6.

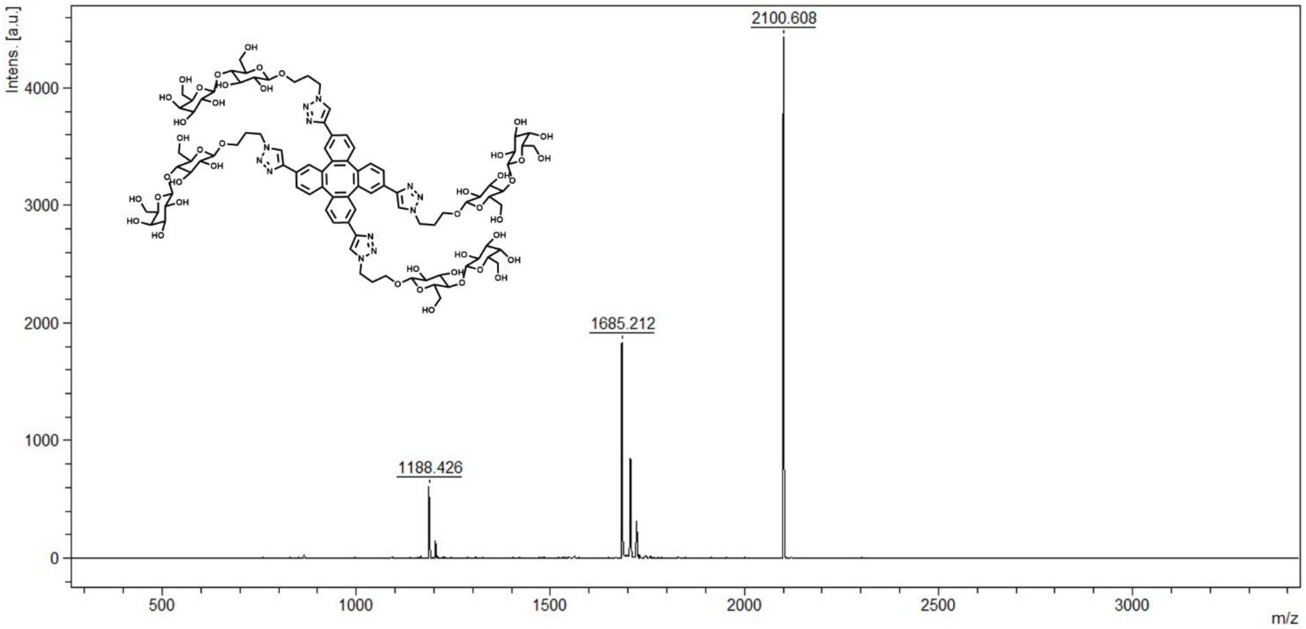

Figure S29. MALDI-TOF-MS of compound **TLTP**.

9. HPLC of TLTP

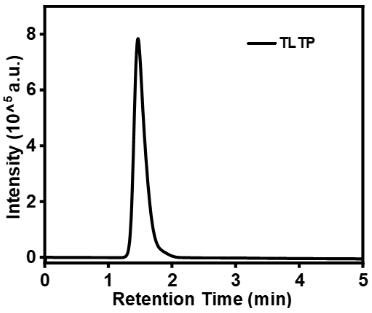

Peak Name	Retention Time (min)	Height	Area	Plates	Tailing	Area %
1	1.465	785629	9981253	321	1.634	99.935
2	2.650	92	1950	1290	0.020	0.020

Chromatographic column： 2.1 * 150mm Column (XTerra® MS C18 5μm)
Mobile phase: H2O/MeOH = 20/80
Flow Rate: 1.0 mL/min
Instrument Model: Shimadzu Prominence LC-20A HPLC

Figure S30. HPLC of compound **TLTP**.

10. Cartesian coordinates and corresponding energies for DFT Calculation

Calculation 1

Structure of TLTP-Dimer
Method: #p opt=(cartesian,loose) b3lyp/6-31g(d) em=gd3bj
Total Energy (Hartree)=-15094.0975
Cartesian coordinates:
0 1

C	-0.63630100	1.72529000	0.09406400
C	-1.35315200	1.53139100	-1.10701800
C	1.98307000	1.79714100	-1.42499600
C	3.11284500	1.00147700	-1.20975300
H	3.64234600	1.09276800	-0.26992000
C	-1.14379000	2.36621900	-2.32331100
C	0.09251100	2.49981500	-2.99124300
C	0.38102500	2.79504500	0.27069900
C	1.60788300	2.82714800	-0.41716100
C	-0.99235800	0.98059700	1.22713300
H	-0.44238800	1.13698500	2.14968000
C	-2.01742400	0.04499300	1.18335300
H	-2.27533400	-0.51472500	2.07615700
C	-2.26568900	3.00594600	-2.86419800
H	-3.21802800	2.89152200	-2.35709200
C	2.51674200	3.85894200	-0.15444600
H	3.44729500	3.88080700	-0.71524400
C	1.29149600	1.69456400	-2.64658400
C	2.23325400	4.87169800	0.77070600
C	2.89752200	0.02311800	-3.39807300
H	3.21899200	-0.68178800	-4.15929000
C	-2.19590300	3.78601200	-4.02202200
C	0.12004800	3.79287200	1.22159100
H	-0.81901700	3.75751300	1.76485500
C	-2.39688400	0.59814900	-1.12717900
H	-2.95845300	0.46125100	-2.04095100
C	0.16368800	3.31266000	-4.13344200
H	1.12173100	3.41832300	-4.63476000
C	1.79305600	0.82972700	-3.62926400
H	1.27937500	0.78382200	-4.58364500
C	-0.95254300	3.96166900	-4.64136900
H	-0.88264700	4.58509700	-5.52618400
C	1.01899400	4.81951300	1.46839700
H	0.79167400	5.59567700	2.19020600
C	3.56718400	0.08801200	-2.17015400
C	4.72010700	-0.77762500	-1.91615400
C	5.50615300	-1.48037600	-2.81806500
N	5.20635700	-1.04107700	-0.66497800
N	6.42208900	-2.12805000	-2.06423800
H	5.48868300	-1.56036600	-3.89274500
N	6.22820900	-1.85785300	-0.76026900
C	7.59777700	-2.87931800	-2.51253700
C	8.83738300	-1.97803600	-2.54412500
H	7.72330000	-3.71940300	-1.82505100
H	7.35002500	-3.28677200	-3.49739300
C	10.08121900	-2.65921300	-3.12196100
H	9.05977300	-1.67110100	-1.52224900
H	8.62219500	-1.06967200	-3.11937300
O	11.22243500	-1.81915600	-2.90133400
H	10.23882000	-3.65110900	-2.68155100
H	9.99296400	-2.78813400	-4.20670200
C	12.04812300	-2.22145300	-1.85822600
H	12.49591500	-3.20563700	-2.06634400
C	13.14215900	-1.16618700	-1.67555000
O	11.23044500	-2.31628300	-0.68765600
H	12.63894400	-0.19769200	-1.60370800
O	14.00090400	-1.21986000	-2.79113800
C	13.89413400	-1.39109900	-0.34776500
C	11.91389700	-2.59638400	0.53425500

H	14.38071100	-0.32513100	-2.89652300
H	14.44986200	-2.33911100	-0.39728000
O	14.81533300	-0.31336100	-0.16075600
C	12.87891600	-1.44853100	0.78331100
H	12.47776100	-3.53597700	0.44656900
C	10.83090800	-2.71914000	1.59784300
H	15.07948000	-0.25440100	0.77962900
H	12.32736400	-0.50624400	0.81410400
O	13.51613300	-1.65080600	2.05779300
H	10.30449900	-1.76744900	1.69342900
H	11.26527900	-2.96861100	2.56550200
O	9.90845700	-3.74250700	1.25697500
C	13.59154700	-0.54458100	2.91038100
H	9.56394600	-3.50311700	0.38103100
H	12.83494700	0.20195700	2.64332800
C	13.39336000	-1.03484900	4.35423200
O	14.88971500	0.01547200	2.67913400
O	12.05065700	-1.43372500	4.53208200
H	14.00165200	-1.93166400	4.46511000
C	13.89315600	-0.03036800	5.40509100
C	15.21214300	1.08876200	3.58015600
H	11.50305800	-0.63360900	4.67821500
O	13.97411800	-0.63271300	6.68034500
H	13.17552600	0.79735900	5.48483900
C	15.26963800	0.52863500	4.99999800
H	14.41091200	1.84368800	3.53066600
C	16.50931000	1.73067200	3.10813900
H	14.86066600	-1.03930100	6.70556100
O	16.27639500	-0.46264200	5.16689700
H	15.53331400	1.33108200	5.69731600
O	16.33675100	2.49627300	1.92267800
H	16.90547800	2.35668000	3.92037200
H	17.24753300	0.95569200	2.89155200
H	16.16725500	-1.17313100	4.50012100
H	15.59699900	3.12271700	2.05901400
C	3.15298200	5.98758600	1.00527400
C	4.46387000	6.18080200	0.60200900
N	2.77431100	7.09441600	1.72387900
N	4.80061500	7.40045500	1.09997800
H	5.15054400	5.56607700	0.04073800
N	3.76127100	7.94164800	1.77668700
C	6.05062400	8.13589800	0.97709400
C	6.27807600	8.71074700	-0.44034500
H	5.98746600	8.93879300	1.71415700
H	6.86722500	7.47541900	1.27430300
C	7.75836100	8.82547800	-0.83240900
H	5.81664100	9.70228100	-0.49877600
H	5.76380000	8.09480200	-1.18654800
O	8.29554800	7.65767700	-1.47380200
H	7.89105600	9.61719900	-1.57302700
H	8.36959900	9.07516400	0.04559000
C	8.06021400	6.45130600	-0.80516300
H	6.99315200	6.31411100	-0.63116500
C	8.51875400	5.26822000	-1.67013300
O	8.64805100	6.45115800	0.50214000
H	8.16365300	5.44249200	-2.69488100
O	7.87435400	4.17491300	-1.05483500
C	10.07037700	5.11970500	-1.74764800
C	9.91439900	5.80208500	0.59260700
H	8.33209700	3.32374500	-1.23197900
H	10.30388200	4.09539600	-1.43632500
O	10.49206900	5.30863100	-3.09055100
C	10.68721700	6.05383100	-0.70921000
H	9.72961200	4.72502700	0.69714400
C	10.66429200	6.25615400	1.84692500
H	11.41573900	5.63433500	-3.12158300
H	10.54118100	7.09694600	-1.00812900
O	12.07468900	5.83114300	-0.45830600
H	9.93506500	6.50630700	2.62694000

H	11.28985700	7.12899200	1.65322000
O	11.56153600	5.22987000	2.29958500
C	12.96061600	6.69841100	-1.08639600
H	11.03678400	4.54494700	2.74208900
H	12.53954300	7.71551100	-1.15256100
C	14.24729500	6.74175500	-0.26134500
O	13.15670000	6.18356800	-2.39158500
O	14.01747600	7.72529000	0.74393900
H	14.36695100	5.76528900	0.21403900
C	15.53128700	7.04992900	-1.06574400
C	14.10882600	6.91438400	-3.16404600
H	14.75723500	7.66787200	1.36939900
O	16.65005400	6.44700300	-0.42818400
H	15.68217000	8.13270900	-0.99446200
C	15.50578500	6.69844400	-2.57917100
H	13.85907300	7.98918000	-3.11566800
C	13.96793200	6.41090000	-4.59936000
H	16.42677300	5.50543400	-0.25554300
O	15.85712900	5.35450700	-2.83154500
H	16.21331100	7.37441000	-3.08611700
O	12.61878800	6.41114600	-5.04023500
H	14.62223100	7.00316900	-5.25545000
H	14.29772600	5.37262900	-4.64268200
H	16.55619800	5.09706200	-2.20700300
H	12.31194800	7.32859300	-5.11261800
C	-3.39708200	4.40289700	-4.58330100
C	-4.71847600	4.00577500	-4.47818000
N	-3.35449700	5.52923400	-5.36455100
N	-5.40238900	4.92079000	-5.20777600
H	-5.18314900	3.13420200	-4.04463900
N	-4.56363600	5.84903900	-5.73166200
C	-6.83640100	5.11630900	-5.33380500
C	-7.34311600	6.10376500	-4.27619300
H	-7.01504700	5.50344900	-6.34113400
H	-7.32073900	4.13897600	-5.24972900
C	-8.76952700	6.57932500	-4.52421000
H	-6.68407500	6.97867300	-4.28845300
H	-7.26943600	5.65284300	-3.27852200
O	-9.10311000	7.58453400	-3.55793300
H	-8.85696800	7.04731700	-5.51039200
H	-9.49514600	5.75732200	-4.47849900
C	-9.93044500	7.18176200	-2.52126300
H	-9.77270800	6.11772700	-2.27148900
C	-9.61105200	8.04880300	-1.30816500
O	-11.28336800	7.38886300	-2.89688300
H	-9.75855600	9.09916500	-1.59870800
O	-8.29820700	7.82559000	-0.81949100
C	-10.55977700	7.68759700	-0.18106200
C	-12.22390900	6.98106500	-1.88598000
H	-7.68530100	8.05499100	-1.53697100
H	-10.39068000	6.63582200	0.09050200
O	-10.32647700	8.52089000	0.95725300
C	-12.00839400	7.83612000	-0.62146300
H	-12.07203800	5.92575700	-1.62629700
C	-13.59336100	7.13144200	-2.53987700
H	-10.85558900	6.59538400	4.01579100
H	-12.22291200	8.89260500	-0.82542500
O	-12.80612400	7.36584100	0.46074000
H	-13.80257200	8.20453200	-2.69317500
H	-14.36483000	6.71500000	-1.89003500
O	-13.64517900	6.41637200	-3.75728000
C	-14.03490400	8.00972900	0.70323800
H	-12.85099100	6.68737000	-4.24744600
H	-14.50975700	8.29270200	-0.24009800
C	-14.91022600	6.99455800	1.43243700
O	-13.88930100	9.23699400	1.39305800
O	-15.68689800	6.27384400	0.47798200
H	-14.25305900	6.29727900	1.95784600
C	-15.79091200	7.69167900	2.45962500

C	-13.91826700	9.32392100	2.82979400
H	-16.45025200	5.93412900	0.98482900
O	-16.79161400	6.81747000	2.94636500
H	-16.33176300	8.51769800	1.98462400
C	-14.84633200	8.28039400	3.51685400
H	-14.36057600	10.31065000	3.00908400
C	-12.49858200	9.35994900	3.40005000
H	-16.39023500	6.12323500	3.51156300
O	-14.11682100	7.24889200	4.20931200
H	-15.43511900	8.78423800	4.28913900
O	-11.80903400	8.12521000	3.20050300
H	-11.94675100	10.17605200	2.92239600
H	-12.54038000	9.54528900	4.47795200
H	-14.82060800	5.73182200	4.63286200
H	-11.39611200	8.15698600	2.30383300
C	-3.77169900	-1.19204500	-0.08504600
C	-3.82729500	-2.44042200	0.51945300
N	-4.86420500	-1.08164300	-0.89948200
N	-4.94283000	-3.02129500	0.02037400
H	-3.15949300	-2.95534600	1.18746500
N	-5.56494400	-2.18707300	-0.84015400
C	-5.52791300	-4.33033300	0.30605700
C	-6.98030100	-4.15483700	0.75698400
H	-5.44990400	-4.95066100	-0.59260300
H	-4.90977200	-4.79076200	1.08154200
C	-7.64320700	-5.45114500	1.20700200
H	-7.55765900	-3.73759700	-0.07358700
H	-7.01615400	-3.44028200	1.58281500
O	-9.04975500	-5.26807000	1.42533500
H	-7.57454100	-6.22772700	0.43843000
H	-7.18730300	-5.83818900	2.12742800
C	-9.51058100	-4.85169100	2.71646300
H	-8.87410000	-5.30941200	3.47973900
C	-9.46805100	-3.32431700	2.86893700
O	-10.79113400	-5.40209000	2.89102400
H	-9.52910800	-2.87208900	1.87205100
O	-8.22968600	-3.01902400	3.47996000
C	-10.67787000	-2.80745200	3.66009200
C	-11.95108100	-4.66494900	2.46234400
H	-8.01847600	-2.08792100	3.24946400
H	-10.71825900	-3.27572300	4.65313500
O	-10.51526900	-1.40061200	3.77986400
C	-11.91809000	-3.17121500	2.84399900
H	-12.77153100	-5.12658500	3.01239100
C	-12.29228500	-4.78045300	0.96741300
H	-11.35988700	-0.98359900	4.05626200
H	-11.89591300	-2.56551300	1.93496400
O	-13.16382900	-2.91791900	3.51368800
H	-13.09482200	-4.05834500	0.81086600
H	-14.86047200	-5.45926900	0.76044400
O	-11.24560200	-4.37555000	0.12322600
C	-13.77626200	-1.69615300	3.25145800
H	-10.38322100	-4.74143300	0.42717200
H	-13.63968900	-1.40364500	2.19891200
C	-15.27201000	-1.82919000	3.56893000
O	-13.14959600	-0.71487700	4.09212900
O	-15.93097100	-2.53283200	2.54759000
H	-15.34628500	-2.34541900	4.54075200
C	-15.97162800	-0.45773900	3.68832200
C	-13.70581900	0.59870300	3.90041300
O	-17.24148700	-0.57623300	4.29638300
H	-16.13935400	-0.09012700	2.66938400
C	-15.12190700	0.56471500	4.45646400
H	-13.74598100	0.82489900	2.82457700
C	-12.76738200	1.58490800	4.57643700
H	-17.05870400	-0.59066600	5.25330300
O	-15.11023300	0.24067000	5.85180700
H	-15.59280600	1.54789100	4.37520200
O	-11.51992100	1.63848800	3.90923100

H	-13.25537800	2.56679100	4.61470600
H	-12.57038700	1.26694700	5.60303900
H	-14.45448900	-0.47262200	5.95090800
H	-11.69138800	1.92479600	2.99060500
C	-0.92215500	-4.36880600	-0.18456400
C	-1.58741500	-4.28715600	-1.43031300
C	1.65552900	-3.37612100	-1.44888000
C	2.91655900	-3.97277100	-1.33989800
H	3.40300400	-4.01434800	-0.37204400
C	-1.50053100	-3.13253600	-2.36839500
C	-0.29542900	-2.67534900	-2.93679200
C	-0.08398700	-3.28868400	0.39731900
C	1.08354500	-2.78650100	-0.20827100
C	-1.16538400	-5.49046100	0.62841700
H	-0.64135200	-5.56146000	1.57647600
C	-2.05764100	-6.48876800	0.26482400
H	-2.22682100	-7.32826900	0.93353500
C	-2.70586200	-2.55632200	-2.79398200
H	-3.63180900	-2.92292100	-2.36901600
C	1.83557300	-1.81068400	0.45304500
H	2.73297600	-1.43990600	-0.02469100
C	1.02013400	-3.32556200	-2.70346000
C	1.46512900	-1.31271800	1.70609900
C	2.94110700	-4.45725500	-3.69581500
H	3.42024400	-4.87641700	-4.57674000
C	-2.75047900	-1.50852200	-3.71934700
C	-0.41352600	-2.82380300	1.68153000
H	-1.27904900	-3.24502300	2.18461700
C	-2.47968100	-5.30737900	-1.78515600
H	-3.00410300	-5.24367400	-2.73147500
C	-0.35085700	-1.64062800	-3.88177900
H	0.57738400	-1.30969400	-4.32646700
C	1.69454300	-3.85650700	-3.81534000
H	1.21161800	-3.80729000	-4.78663200
C	-1.54372100	-1.03829800	-4.25291000
H	-1.55629500	-0.21040800	-4.95374000
C	0.33507800	-1.85308200	2.32964300
H	0.04854100	-1.49443800	3.31231200
C	3.56367000	-4.53857300	-2.44297300
C	4.83998500	-5.23257200	-2.26206000
C	5.56994700	-6.01143700	-3.14947600
N	5.50559400	-5.23924800	-1.06651600
N	6.63555300	-6.44777400	-2.43902400
H	5.41086300	-6.29510000	-4.17768400
N	6.58412200	-5.96595200	-1.17686100
C	7.75640200	-7.28479300	-2.85413300
C	8.51422300	-6.68565300	-4.04604400
H	8.40175000	-7.35926000	-1.97859700
H	7.37641600	-8.28401800	-3.09204000
C	9.98403700	-7.11204000	-4.09774000
H	8.49700900	-5.59240800	-3.98135300
H	8.02296900	-6.96139200	-4.98774400
O	10.78739900	-6.31346100	-3.23441400
H	10.10109500	-8.17704500	-3.85307200
H	10.37594200	-6.95563600	-5.10789700
C	11.21935700	-6.86416700	-2.03495600
H	11.45587800	-7.93583200	-2.15374000
C	12.44212900	-6.08150700	-1.58338100
O	10.18793500	-6.70243500	-1.06652400
H	12.12700000	-5.03028300	-1.53854900
O	13.49564700	-6.26092500	-2.50638800
C	12.86820000	-6.49057300	-0.17876400
C	10.50061300	-7.23522500	0.23016700
H	14.28132300	-5.88227200	-2.07656300
H	13.25084300	-7.52381300	-0.20297900
O	13.91626700	-5.58711300	0.16483500
C	11.68205000	-6.42710500	0.77752300
H	10.76388800	-8.30039000	0.13377300
C	9.25244300	-7.09202700	1.09847300

H	14.12781500	-5.73135300	1.11072600
H	11.33445300	-5.39551200	0.86640400
O	12.04251400	-6.91608600	2.08445400
H	9.02942200	-6.02559500	1.21894000
H	9.48080200	-7.49482400	2.09045600
O	8.15470300	-7.79952000	0.54644600
C	12.39338900	-5.93710200	2.98925900
H	7.58477400	-7.14359400	0.10347000
H	11.87875600	-4.99559700	2.76250100
C	12.10842400	-6.33930500	4.42601100
O	13.82733000	-5.70190900	2.89007100
O	10.72674200	-6.57251900	4.58248900
H	12.68924000	-7.25185600	4.64336300
C	12.57029000	-5.20249300	5.36220700
C	14.20623600	-4.50357300	3.59633300
H	10.56901300	-6.56375300	5.54265400
O	12.39131800	-5.60393200	6.71200500
H	11.91513200	-4.33784700	5.20213000
C	14.02290100	-4.78824700	5.08941700
H	13.53706600	-3.69319600	3.28875500
C	15.63992900	-4.14176300	3.18017600
H	13.18671200	-6.12501500	6.92748300
O	14.90556400	-5.82831900	5.52391900
H	14.26935400	-3.90861900	5.69162000
O	15.89796700	-2.74389600	3.29673700
H	16.36297200	-4.65141800	3.81971200
H	15.79717700	-4.47106700	2.14517700
H	14.87063400	-6.51334200	4.83281000
H	15.35209300	-2.29576000	2.62562700
C	2.23512400	-0.25283300	2.35606400
C	3.54997200	0.13333800	2.16524400
N	1.69325200	0.56261500	3.31774000
N	3.72640800	1.16608400	3.02753600
H	4.32463100	-0.24557000	1.51287900
N	2.58998100	1.41951300	3.71894200
C	4.91969300	1.96797100	3.24877700
C	5.25177100	2.87425400	2.04853600
H	4.71607800	2.56019300	4.14324900
H	5.74578400	1.29047500	3.48370800
C	6.73411400	3.25115000	1.93968100
H	4.63626300	3.77617600	2.11145100
H	4.97141400	2.37952300	1.11290800
O	7.45948100	2.38791800	1.06060700
H	6.85395600	4.23611000	1.48582400
H	7.21688400	3.26668100	2.92753600
C	7.67398700	1.06947700	1.47456700
H	6.75290200	0.57866700	1.79932100
C	8.25475000	0.33376400	0.27002900
O	8.53831600	1.00838500	2.60250600
H	7.54784000	0.42413600	-0.55894200
O	8.47394600	-1.01912600	0.62462300
C	9.58694900	0.96431700	-0.19093200
C	9.94057900	1.07506100	2.32305400
H	7.65628600	-1.51374400	0.39610800
H	10.23943500	0.14108400	-0.49314300
O	9.31663400	1.81053500	-1.30442100
C	10.26201700	1.72464600	0.97172900
H	10.33939000	0.05391200	2.31250400
C	10.57112300	1.80246800	3.50085000
H	10.05728400	1.80703500	-1.95009500
H	9.90405200	2.75777100	0.95753700
O	11.69575400	1.71484200	0.91344500
H	10.05959300	2.77003100	3.63798500
H	11.63078600	1.98607200	3.31991200
O	10.48089200	1.02502800	4.69245000
C	12.27830000	2.55078300	-0.03631100
H	9.54505700	0.77706900	4.79200200
H	11.75663900	3.51474600	-0.06163000
C	13.74177700	2.77693200	0.34664400

O	12.16416100	1.92621300	-1.29751100
O	13.82947600	3.56642300	1.53820300
H	14.17089600	1.80773700	0.60082700
C	14.52046500	3.40447100	-0.82820800
C	12.78622400	2.66924400	-2.35764600
H	13.14168700	4.26689800	1.54797400
O	15.89073900	3.64384500	-0.51125100
H	14.09487500	4.38367000	-1.01714900
C	14.30528900	2.66316800	-2.15918700
H	12.46934600	3.71773300	-2.28989300
C	12.20768400	2.12308900	-3.65017200
H	16.23464300	2.94774600	0.08398000
O	14.90800800	1.38470800	-2.24166800
H	14.76401500	3.27632800	-2.93862600
O	10.80085800	2.36800000	-3.67755400
H	12.71190000	2.59241000	-4.50602900
H	12.34187600	1.04066500	-3.71688700
H	14.89826100	0.92539700	-1.37340100
H	10.66555300	3.33954500	-3.71131600
C	-4.02018000	-0.87952100	-4.09241200
C	-5.31381900	-1.37061000	-4.03975900
N	-4.08621400	0.41679100	-4.53162200
N	-6.09066100	-0.34200300	-4.45133200
H	-5.71991700	-2.32097000	-3.73403100
N	-5.33283300	0.74242100	-4.74164400
C	-7.54289700	-0.22484100	-4.52443100
C	-8.13334400	0.40395100	-3.25704800
H	-7.75585200	0.39515700	-5.39788400
H	-7.95165000	-1.22287700	-4.71035600
C	-8.18012800	-0.56720300	-2.08610600
H	-9.14035300	0.77830100	-3.47785100
H	-7.51786400	1.26390100	-2.97351400
O	-8.34839600	0.20665500	-0.89949900
H	-9.01677100	-1.27167400	-2.19995500
H	-7.25371100	-1.14165900	-2.00878800
C	-8.49404200	-0.54502700	0.27248700
H	-7.80236900	-1.39055200	0.28569900
C	-8.23700300	0.39184700	1.45282500
O	-9.78808300	-1.13879200	0.36411900
H	-7.24165100	0.82604100	1.34323800
O	-8.24560600	-0.34530100	2.67200700
C	-9.26728400	1.54300300	1.48245700
C	-10.88886300	-0.24428600	0.61352300
H	-9.16469300	-0.47634600	2.99475300
H	-9.61266300	1.62223600	2.52150700
O	-8.63138700	2.73948700	1.07231500
C	-10.48611700	1.23290100	0.59691900
H	-11.27101700	-0.47073000	1.61491100
C	-11.96094800	-0.55786800	-0.42497300
H	-9.32095800	3.43009200	1.04009300
H	-10.24659100	1.54403900	-0.42331200
O	-11.65279100	1.95249100	1.04737400
H	-11.54839400	-0.35581900	-1.42615500
H	-12.82540800	0.09218100	-0.26952500
O	-12.43526700	-1.89143700	-0.32535600
C	-11.90414800	3.18841900	0.40808500
H	-11.67603100	-2.50698900	-0.35684600
H	-11.62265500	3.12120900	-0.64705300
C	-13.40751800	3.45321200	0.52091000
O	-11.09703800	4.22263000	0.93044400
O	-13.70948300	4.48778100	-0.39505200
H	-13.92918400	2.52345300	0.25062700
C	-13.72676700	3.78467100	1.95179100
C	-11.52492000	4.90628500	2.13863600
H	-14.51920100	4.96271200	-0.11008700
O	-14.96410700	3.38336900	2.35406300
H	-9.36580700	8.50118600	1.11395600
C	-12.81839800	4.41584800	2.70360900
H	-11.65617900	5.94532000	1.82564900

C	-10.40728700	4.86790600	3.19242400
H	-15.15850300	3.80036600	3.22517700
O	-15.42317400	4.94526100	4.57793300
H	-12.98591100	4.65657600	3.74581700
O	-10.76700400	5.66931900	4.30757600
H	-10.27726900	3.85162900	3.57181400
H	-9.46075300	5.19400900	2.73481400
H	-15.74507200	4.76279000	5.47350800
H	-13.21274200	7.18569400	3.83009800
C	-3.76484900	-7.37419200	-1.33476300
C	-4.16106300	-8.54819500	-0.71842900
N	-4.58558000	-7.17926100	-2.41812400
N	-5.19220500	-9.00361500	-1.47354900
H	-3.80715900	-9.07847600	0.15166000
N	-5.45047400	-8.15153000	-2.49178600
C	-6.19775500	-9.99926300	-1.11900300
C	-7.29753000	-9.30235400	-0.31736500
H	-6.58012400	-10.41795600	-2.05371500
H	-5.70563100	-10.79917400	-0.55777300
C	-8.48547900	-10.16656300	0.09063500
H	-7.67877400	-8.47357700	-0.91891200
H	-6.86008100	-8.87046100	0.59102500
O	-9.40286600	-9.32374900	0.78559100
H	-8.95929100	-10.63349200	-0.78153900
H	-8.18795700	-10.95956900	0.78640700
C	-10.50322900	-8.88495200	0.05445000
H	-11.23028800	-9.70412500	-0.09317900
C	-11.11427600	-7.70171100	0.79902600
O	-10.07232100	-8.42449100	-1.22624800
H	-10.30470300	-6.97313700	0.87563600
O	-11.55995400	-8.09021100	2.08014700
C	-12.27322600	-7.11316800	-0.02170200
C	-11.15650600	-8.03837800	-2.07559600
H	-11.31179900	-7.36263500	2.67528700
H	-13.07339400	-7.86056600	-0.05582100
O	-12.90608300	-6.02306400	0.65193300
C	-11.86091800	-6.81911800	-1.47553800
H	-11.86842500	-8.87298200	-2.16717600
C	-10.54832000	-7.74278000	-3.44085700
H	-11.17061800	-5.97347600	-1.50505700
O	-12.97109400	-6.55198900	-2.33254100
H	-9.85821100	-6.88922700	-3.34001000
H	-11.33851500	-7.45475600	-4.13625700
O	-9.89362200	-8.87997700	-3.97290200
C	-13.50440900	-5.27097900	-2.27227900
H	-9.20507300	-9.11255000	-3.32982400
H	-12.77946000	-4.56191300	-1.85103500
C	-13.94587400	-4.81041800	-3.65440000
O	-14.66637000	-5.31979300	-1.44520000
O	-12.81384200	-4.68251300	-4.49412900
H	-14.63796200	-5.57340600	-4.04657700
C	-14.68940600	-3.46507600	-3.54894400
C	-15.21017500	-4.01616400	-1.18470100
H	-13.13608200	-4.21510900	-5.28420000
O	-15.19268100	-3.10488900	-4.82884300
H	-13.95821800	-2.69673000	-3.26955400
C	-15.80849600	-3.50879000	-2.49595300
H	-14.40823100	-3.33097400	-0.87315800
C	-16.17052200	-4.22190200	-0.02418900
H	-16.03401700	-3.59201500	-4.90822600
O	-16.86355800	-4.35530700	-2.96225500
H	-16.24365300	-2.51135900	-2.37855200
O	-15.47709200	-4.77547100	1.09235300
H	-16.58825800	-3.26751600	0.30297500
H	-16.99355400	-4.87894600	-0.33433200
H	-16.57898500	-5.26421400	-2.75788500
H	-15.53804100	-3.41878900	2.41284400
C	-2.72709000	-0.16588100	-0.00234300
C	-2.74400700	-6.39895100	-0.95308200

Calculation 2

Structure of TLTP-Monomer

Method: #p opt=(cartesian,loose) b3lyp/6-31g(d) em=gd3bj

Total Energy (Hartree)= -7547.4210

Cartesian coordinates：

0 1

C	1.22314900	-1.78073100	-1.10901600
C	1.63316100	-1.78261900	0.23911400
C	-1.63310500	-1.78260800	-0.23786900
C	-2.61183900	-2.68635500	-0.65880300
H	-2.93098600	-2.68545400	-1.69525800
C	1.10668200	-0.79557600	1.22220900
C	-0.23973800	-0.79806500	1.63541200
C	0.23980200	-0.79806900	-1.63415800
C	-1.10661700	-0.79555800	-1.22095900
C	1.81608400	-2.69204100	-1.99330900
H	1.50436600	-2.68777800	-3.03365200
C	2.78121200	-3.59484100	-1.56339100
H	3.21645200	-4.29069500	-2.27542100
C	1.99754100	0.11156800	1.80076600
H	3.03382900	0.08826100	1.47610000
C	-1.99746000	0.11161200	-1.79949700
H	-3.03374100	0.08833600	-1.47480600
C	-1.22309600	-1.78071100	1.11026000
C	-1.58755100	1.03173600	-2.77200300
C	-2.78116700	-3.59481500	1.56463700
H	-3.21643300	-4.29064900	2.27666900
C	1.58764900	1.03168700	2.77328400
C	0.64326000	0.11304500	-2.62025500
H	1.68056000	0.10640500	-2.94275700
C	3.19308400	-3.60441100	-0.22389400
C	2.61190000	-2.68636700	0.66004300
H	2.93105100	-2.68546400	1.69649700
C	-0.64317500	0.11303100	2.62153100
H	-1.68047400	0.10640400	2.94403400
C	-1.81604200	-2.69201200	1.99455700
H	-1.50433300	-2.68774500	3.03490200
C	0.24672200	1.02124400	3.18140600
H	-0.07996700	1.72847800	3.93562400
C	-0.24662100	1.02128300	-3.18011500
H	0.08008100	1.72853300	-3.93431100
C	-3.19302400	-3.60439900	0.22513500
C	-4.21017900	-4.54008400	-0.25869500
C	-4.86336400	-5.57085500	0.39563900
N	-4.67743400	-4.50931200	-1.54831700
N	-5.68320200	-6.10820600	-0.54152700
H	-4.80536700	-5.95829600	1.40056000
N	-5.56528000	-5.44759300	-1.71658900
C	-6.67216100	-7.16536900	-0.40283300
C	-8.02229700	-6.62665200	0.07753500
H	-6.76573700	-7.62937200	-1.38797600
H	-6.27221900	-7.91356200	0.28922100
C	-9.05997300	-7.73359400	0.18198300
H	-8.37323200	-5.86388200	-0.62516500
H	-7.90548100	-6.14312500	1.05450000
O	-10.28337900	-7.14143500	0.60778700
H	-9.20201900	-8.22810500	-0.78718800
H	-8.74728400	-8.50164700	0.90782300
C	-11.30245500	-8.05273800	0.87715600
H	-10.94232600	-8.83778600	1.56871300
C	-12.44523500	-7.27185100	1.52629200
O	-11.72245200	-8.65693000	-0.33426600
H	-12.74642700	-6.48213400	0.82068400
O	-12.01765000	-6.73023300	2.76177500
C	-13.64331400	-8.18472000	1.77323100
C	-12.72597100	-9.65293100	-0.10911600
H	-11.23361000	-6.19531500	2.55577800

H	-13.37526400	-8.89288200	2.57174500
O	-14.73793300	-7.37565900	2.15660400
C	-13.96313000	-8.99592300	0.50409200
H	-12.33711100	-10.41736200	0.58185500
C	-12.99813000	-10.29801600	-1.46128800
H	-15.37946600	-7.94410500	2.62179000
H	-14.40708100	-8.31009800	-0.23131100
O	-14.87371700	-10.06722100	0.77027700
H	-13.40763700	-9.53150100	-2.13977400
H	-13.74553600	-11.08572900	-1.35000900
O	-11.82110700	-10.88375800	-1.98956800
C	-16.21954300	-9.76540000	0.76499200
H	-11.15296600	-10.17914100	-1.97448600
H	-16.42733500	-8.90925100	0.10065100
C	-17.02797600	-10.97965300	0.33002200
O	-16.61171800	-9.43112400	2.09896000
O	-16.67529600	-11.31754200	-0.99732900
H	-16.80028800	-11.79515400	1.03223100
C	-18.52577600	-10.64371700	0.40172900
C	-17.95393500	-8.98582000	2.19171400
H	-17.35215300	-11.95039100	-1.29318400
O	-19.30008100	-11.77647600	0.03967700
H	-18.73107100	-9.87745600	-0.35527000
C	-18.92697700	-10.10298700	1.77826700
H	-18.11138000	-8.13880000	1.49612600
C	-18.14799500	-8.49483800	3.62178100
H	-19.33135200	-12.32488600	0.84486700
O	-18.88198200	-11.22785100	2.66140500
H	-19.94926600	-9.70197200	1.72177100
O	-17.20092700	-7.50961700	3.99264700
H	-19.18423200	-8.13970500	3.74696400
H	-17.98238300	-9.33120700	4.30577700
H	-19.45817000	-11.05723300	3.42012200
H	-17.29932700	-6.74167600	3.40675000
C	-2.52977600	1.98712100	-3.35693800
C	-3.87636900	2.18455400	-3.10297300
N	-2.15561900	2.88595800	-4.32327600
N	-4.23956800	3.18887100	-3.93808500
H	-4.57545000	1.70971300	-2.43275900
N	-3.18082200	3.61126400	-4.66875400
C	-5.51355200	3.88204900	-4.04151800
C	-5.66622800	4.95738300	-2.96265400
H	-5.53796900	4.32357900	-5.04089100
H	-6.31368900	3.13704500	-3.97793300
C	-6.98165400	5.70810400	-3.10073200
H	-4.83362000	5.66420500	-3.04096300
H	-5.61314500	4.49946100	-1.96800100
O	-7.02934600	6.68849000	-2.06833800
H	-7.05314900	6.19465900	-4.08165300
H	-7.83994100	5.02386400	-3.00201900
C	-8.23458600	7.38138600	-1.98320300
H	-9.08413200	6.67333600	-1.95017800
C	-8.19100900	8.20162100	-0.69390000
O	-8.37346000	8.22149700	-3.11675000
H	-7.31340900	8.86373700	-0.75424000
O	-8.11481100	7.33692600	0.42362500
C	-9.44306700	9.06509800	-0.56711300
C	-9.62744800	8.91153200	-3.11397300
H	-7.32902800	6.78581000	0.27492100
H	-10.29897300	8.39958100	-0.37825800
O	-9.24921400	9.96249700	0.50865300
C	-9.70526800	9.81787400	-1.88450800
H	-10.45048700	8.18035600	-3.08192500
C	-9.69518900	9.67918600	-4.42729200
H	-10.12710100	10.27000900	0.80101400
H	-8.95004300	10.61110100	-1.97625000
O	-11.01362800	10.39724900	-1.91745100
H	-8.88140100	10.42303500	-4.44055800
H	-10.64426200	10.21446700	-4.49259300

O	-9.61226300	8.79915900	-5.53456000
C	-11.16680300	11.63647800	-1.33198700
H	-8.81099600	8.27108900	-5.38524600
H	-10.22137500	12.20464200	-1.36310100
C	-12.27415900	12.41608700	-2.02735000
O	-11.55498500	11.44753600	0.03156300
O	-11.91072400	12.63509000	-3.37641500
H	-13.19653000	11.82244600	-1.94517900
C	-12.46477000	13.76398100	-1.31428400
C	-11.61522500	12.65731600	0.76741900
H	-12.53498000	13.30313800	-3.70840900
O	-13.51917200	14.49515900	-1.92100900
H	-11.55345800	14.35334300	-1.47028000
C	-12.69394100	13.58973400	0.19060400
H	-10.64622100	13.18729700	0.68737600
C	-11.84740400	12.26703600	2.22261100
H	-14.33373300	14.11788600	-1.54126300
O	-14.01380700	13.05180800	0.31922500
H	-12.63322200	14.57334200	0.67831800
O	-10.87296300	11.36035300	2.70533300
H	-11.90458800	13.17939600	2.83909600
H	-12.80073000	11.73740900	2.29673900
H	-14.35693000	13.26480200	1.19874300
H	-9.99790600	11.77721200	2.64839600
C	2.52988900	1.98706400	3.35821000
C	3.87641600	2.18466800	3.10403900
N	2.15581100	2.88571600	4.32475200
N	4.23966600	3.18888600	3.93924800
H	4.57542000	1.71001000	2.43361400
N	3.18100600	3.61106000	4.67017000
C	5.51360700	3.88215200	4.04259400
C	5.66631200	4.95722100	2.96346900
H	5.53789200	4.32394800	5.04185200
H	6.31380500	3.13718800	3.97928200
C	6.98164800	5.70810700	3.10151100
H	4.83363000	5.66398800	3.04150500
H	5.61339000	4.49905300	1.96892000
O	7.02932600	6.68829300	2.06892900
H	7.05300400	6.19486200	4.08234500
H	7.84001600	5.02394000	3.00300900
C	8.23459500	7.38109800	1.98355600
H	9.08409800	6.67299400	1.95058500
C	8.19094700	8.20110300	0.69410500
O	8.37362400	8.22140400	3.11694600
H	7.31336900	8.86325300	0.75439700
O	8.11463300	7.33620100	-0.42325200
C	9.44301300	9.06452100	0.56705700
C	9.62763900	8.91139000	3.11392100
H	7.32880000	6.78519100	-0.27442400
H	10.29888400	8.39895200	0.37822800
O	9.24908300	9.96175100	-0.50884200
C	9.70536800	9.81751500	1.88429600
H	10.45064800	8.18017600	3.08191900
C	9.69554000	9.67926300	4.42710300
H	10.12695200	10.26915600	-0.80136800
H	8.95017900	10.61078200	1.97597400
O	11.01375000	10.39685200	1.91699700
H	8.88178200	10.42314600	4.44032300
H	10.64464100	10.21451800	4.49222400
O	9.61268600	8.79942300	5.53452500
C	11.16689100	11.63597100	1.33129200
H	8.81139300	8.27134700	5.38536700
H	10.22147100	12.20414600	1.36237100
C	12.27432200	12.41569900	2.02640800
O	11.55496000	11.44677000	-0.03225800
O	11.91101800	12.63495800	3.37547000
H	13.19666800	11.82201800	1.94426100
C	12.46487800	13.76345300	1.31307700
C	11.61514700	12.65641100	-0.76834100

H	12.53526200	13.30312300	3.70725100
O	13.51932300	14.49475000	1.91958100
H	11.55357700	14.35284500	1.46903300
C	12.69392700	13.58892200	-0.19179400
H	10.64615900	13.18642400	-0.68831100
C	11.84718200	12.26586100	-2.22348500
H	14.33386000	14.11737700	1.53988400
O	14.01377300	13.05095500	-0.32042400
H	12.63318100	14.57244300	-0.67968400
O	10.87267100	11.35911600	-2.70594800
H	11.90432700	13.17810900	-2.84013800
H	12.80048900	11.73619900	-2.29761400
H	14.35682600	13.26378000	-1.20001000
H	9.99763500	11.77602300	-2.64903400
C	4.21025600	-4.54007900	0.25993400
C	4.86353900	-5.57076900	-0.39443000
N	4.67741300	-4.50938400	1.54959300
N	5.68333100	-6.10815600	0.54275600
H	4.80562900	-5.95814000	-1.39938400
N	5.56530400	-5.44762000	1.71785500
C	6.67234700	-7.16526300	0.40405800
C	8.02226000	-6.62658800	-0.07698100
H	6.76631200	-7.62888400	1.38934300
H	6.27224600	-7.91376000	-0.28757800
C	9.06000100	-7.73347500	-0.18137100
H	8.37337300	-5.86352300	0.62531100
H	7.90506800	-6.14343500	-1.05408600
O	10.28321200	-7.14136700	-0.60780700
H	9.20241800	-8.22760800	0.78793800
H	8.74713300	-8.50182900	-0.90681700
C	11.30225200	-8.05268500	-0.87725700
H	10.94195200	-8.83792900	-1.56850300
C	12.44479300	-7.27190000	-1.52694200
O	11.72265200	-8.65654800	0.33419000
H	12.74614600	-6.48197800	-0.82163000
O	12.01680500	-6.73063200	-2.76243800
C	13.64285500	-8.18475600	-1.77400100
C	12.72616800	-9.65254100	0.10899500
H	11.23281700	-6.19567500	-2.55634700
H	13.37460300	-8.89314900	-2.57224600
O	14.73729900	-7.37572500	-2.15792100
C	13.96310000	-8.99560000	-0.50473900
H	12.33715300	-10.41717000	-0.58167000
C	12.99876500	-10.29727100	1.46124700
H	15.37881500	-7.94428700	-2.62299300
H	14.40720500	-8.30954800	0.23035900
O	14.87369900	-10.06689400	-0.77090600
H	13.40842200	-9.53056000	2.13942300
H	13.74618700	-11.08496600	1.34994800
O	11.82193400	-10.88295500	1.99001900
C	16.21950800	-9.76499200	-0.76594700
H	11.15374300	-10.17838600	1.97495200
H	16.42738000	-8.90870400	-0.10180900
C	17.02810600	-10.97910600	-0.33090500
O	16.61139300	-9.43094300	-2.10006000
O	16.67571100	-11.31675900	0.99658200
H	16.80033600	-11.79476000	-1.03291000
C	18.52586800	-10.64308300	-0.40297400
C	17.95356800	-8.98558400	-2.19318300
H	17.35267300	-11.94949700	1.29243100
O	19.30032100	-11.77571600	-0.04084400
H	18.73126300	-9.87665300	0.35382700
C	18.92675800	-10.10261300	-1.77970400
H	18.11111100	-8.13841100	-1.49780400
C	18.14731400	-8.49489700	-3.62339700
H	19.33145500	-12.32429400	-0.84592500
O	18.88164800	-11.22765900	-2.66260100
H	19.94903500	-9.70152800	-1.72349800
O	17.20009600	-7.50984600	-3.99431400

H	19.18350000	-8.13970200	-3.74884200
H	17.98165700	-9.33143200	-4.30717800
H	19.45768800	-11.05717300	-3.42145900
H	17.29846700	-6.74179800	-3.40855100

Calculation 3

Structure of TATP-Trimer

Method：#p opt=(cartesian,loose) b3lyp/6-31g(d,p) em=gd3bj

Total Energy (Hartree)= -3686.9179

Cartesian coordinates：

0 1

C	6.00678600	1.67813700	-0.36812300
C	5.18797800	-0.35486000	-1.65180500
C	5.08336100	-1.43054800	-0.74554400
C	4.18299100	-2.46677300	-1.01518600
H	4.09984500	-3.28751100	-0.31276600
C	3.39233100	-2.46798200	-2.17567000
C	4.43644300	-0.39340900	-2.83517200
H	4.54320900	0.42463400	-3.54082700
C	6.10714500	0.79611900	-1.46303900
C	3.55666300	-1.42971900	-3.10566200
H	2.97822800	-1.43794800	-4.02242900
C	7.02665700	1.08801600	-2.48392200
H	7.10415200	0.40261700	-3.32237700
C	6.80673500	2.82509900	-0.34308300
H	6.71806300	3.51001000	0.49300700
C	7.82769200	2.21965200	-2.44658300
H	8.53420300	2.42322400	-3.24346100
C	7.71704800	3.11544900	-1.37006500
C	5.05118700	1.50064000	0.76115400
C	6.09525600	-0.70875700	1.49670900
C	6.02314000	-1.59281700	0.40080200
C	6.84355000	-2.72572600	0.38623500
H	6.77840800	-3.41139600	-0.45111300
C	7.75009800	-2.99898900	1.42119400
C	7.00819200	-0.98404600	2.52865400
H	7.06416100	-0.29810700	3.36867900
C	5.15829500	0.42908300	1.67108300
C	7.83066200	-2.10083800	2.49997400
H	8.53568400	-2.29254600	3.30300000
C	4.38704000	0.45962200	2.84004600
H	4.49775700	-0.34805900	3.55395100
C	4.12411600	2.51793500	1.00716500
H	4.03983100	3.33216800	0.29926200
C	3.47715300	1.47618500	3.08502100
H	2.87905600	1.47707500	3.98698100
C	3.30357500	2.50219200	2.14444700
C	2.42313800	-3.49088700	-2.41327400
C	1.56693600	-4.31990800	-2.62478000
H	0.80423000	-5.04503200	-2.79912400
C	8.57188800	-4.16521100	1.37557900
C	2.28772500	3.48472700	2.33762700
C	8.50933900	4.30113000	-1.31934700
C	9.28627400	-5.14118000	1.34913900
H	9.90659900	-6.00737500	1.31855600
C	1.38390200	4.27246700	2.50380000
H	0.57126600	4.94797700	2.63871100
C	9.17719300	5.31013500	-1.27791100
H	9.76795600	6.19620900	-1.24157700
C	0.44592300	1.53365600	-0.67499500
C	-0.47673800	-0.64336700	-1.65709400
C	-0.46549000	-1.59476600	-0.61737100
C	-1.29304900	-2.71944400	-0.71282200
H	-1.29010600	-3.43589700	0.09924700
C	-2.13267400	-2.93211500	-1.81504700
C	-1.27881300	-0.89380000	-2.78170500
H	-1.26745300	-0.17622200	-3.59494500
C	0.39848700	0.55274300	-1.68893800

C	-2.09213200	-2.01221500	-2.87362800
H	-2.71778800	-2.17352800	-3.74372200
C	1.16595700	0.75006600	-2.84831400
H	1.10988900	0.00640900	-3.63366600
C	1.30837800	2.62628000	-0.82930200
H	1.35136800	3.36550300	-0.03836300
C	2.01143800	1.83684600	-2.99792100
H	2.60933400	1.95320400	-3.89455200
C	2.11551700	2.78071900	-1.96647200
C	-0.45961300	1.57105200	0.51253400
C	0.41078400	-0.58688000	1.56594800
C	0.46110800	-1.56200700	0.55104000
C	1.31186700	-2.66200000	0.70396900
H	1.35203900	-3.40525500	-0.08218000
C	2.10833100	-2.82913200	1.84652100
C	1.17488800	-0.78975900	2.72635200
H	1.11391800	-0.05225400	3.51655700
C	-0.46893600	0.60567500	1.53994800
C	2.00826900	-1.88413400	2.87739500
H	2.59985900	-2.01216400	3.77650200
C	-1.25905800	0.84030900	2.67596300
H	-1.25825000	0.09517300	3.46165600
C	-1.23829200	2.72595800	0.66335800
H	-1.22422200	3.47751300	-0.11407300
C	-2.03304100	1.98008700	2.81568800
H	-2.63911100	2.13066800	3.70142700
C	-2.02943800	2.94794300	1.80070600
C	-3.04606500	-4.02853000	-1.84353600
C	-3.86012500	-4.92418800	-1.86132800
H	-4.61742400	-5.67554100	-1.87113600
C	3.01064600	-3.92972900	1.95733300
C	-2.80145200	4.14316700	1.93718000
C	3.04957200	3.85509100	-2.06572500
C	3.80211700	-4.84099400	2.04860200
H	4.50470100	-5.63746100	2.12966500
C	-3.45471300	5.15368000	2.06912300
H	-4.03824000	6.03709900	2.17868000
C	3.87939500	4.73348700	-2.14624700
H	4.61594600	5.49997700	-2.21433600
C	-5.20705300	1.51330200	-0.63991600
C	-5.83452900	-0.81301200	-1.48277300
C	-5.75903500	-1.69075300	-0.38128500
C	-6.38685300	-2.93744100	-0.46279100
H	-6.30995100	-3.62050300	0.37509900
C	-7.08729500	-3.34187600	-1.60629300
C	-6.56100800	-1.21334700	-2.61616700
H	-6.62367100	-0.52987600	-3.45813800
C	-5.06842300	0.45504600	-1.56026300
C	-7.18152800	-2.45148800	-2.68884800
H	-7.72996100	-2.74333100	-3.57703600
C	-4.20716400	0.62170700	-2.65457200
H	-4.11998500	-0.18820100	-3.36784500
C	-4.44039600	2.67045200	-0.81220900
H	-4.51481200	3.46737300	-0.08452900
C	-3.46481500	1.77839300	-2.83318700
H	-2.80085000	1.88572500	-3.68301100
C	-3.56269800	2.81923100	-1.89829700
C	-6.27660000	1.52123800	0.40036600
C	-5.27193700	-0.38023400	1.74583700
C	-4.96348000	-1.42364100	0.85124400
C	-3.96594500	-2.34191200	1.19196500
H	-3.74286700	-3.14775300	0.50531200
C	-3.26235300	-2.24964700	2.40241000
C	-4.61348900	-0.33390400	2.98406400
H	-4.87663500	0.45327500	3.68252300
C	-6.33028800	0.62438300	1.48566300
C	-3.62835000	-1.25054200	3.31811000
H	-3.12254900	-1.19127300	4.27523100
C	-7.37326700	0.74599900	2.41697700

H	-7.41036600	0.04969800	3.24809700
C	-7.25692500	2.51263600	0.29147300
H	-7.21229900	3.20847500	-0.53928500
C	-8.34976200	1.72241200	2.29542800
H	-9.15218300	1.79472600	3.02055200
C	-8.29790800	2.62923600	1.22463000
C	-7.66442800	-4.64576700	-1.67497800
C	-8.14287900	-5.75597600	-1.73912000
H	-8.58047700	-6.72626800	-1.79075100
C	-2.17640300	-3.13070900	2.68626000
C	-9.28368600	3.65045300	1.08729200
C	-2.79228900	4.00882300	-2.06730800
C	-1.22692100	-3.84467700	2.91691300
H	-0.36984000	-4.45091000	3.10225700
C	-10.11787800	4.51920900	0.97358300
H	-10.85313300	5.28100100	0.87435100
C	-2.12027100	5.00251700	-2.22307600
H	-1.53360700	5.88218700	-2.35890500

Calculation 4

Structure of TATP-Dimer

Method: #p opt=(cartesian,loose) b3lyp/6-31g(d,p) em=gd3bj

Total Energy (Hartree)= -2457.9369

Cartesian coordinates:

0 1

C	-3.26047500	-1.75372200	-0.63538600
C	-2.49589800	0.45304300	-1.63882700
C	-2.59494400	1.44178200	-0.63996100
C	-1.80065300	2.58831400	-0.72797700
H	-1.86871600	3.33544800	0.05385900
C	-0.90548600	2.78125200	-1.79144000
C	-1.62789200	0.67034100	-2.71862500
H	-1.57159700	-0.08349400	-3.49734000
C	-3.33642700	-0.77163200	-1.64446500
C	-0.83880700	1.80897700	-2.80214100
H	-0.15797400	1.95345600	-3.63383500
C	-4.17758200	-0.99458300	-2.74556300
H	-4.23077400	-0.23554100	-3.51948900
C	-4.02284400	-2.91910200	-0.76235500
H	-3.95990200	-3.67663200	0.01073000
C	-4.94034700	-2.14817000	-2.85982600
H	-5.59193300	-2.29737400	-3.71358600
C	-4.86894100	-3.13322400	-1.86085600
C	-2.35887700	-1.64803700	0.54676000
C	-3.58417400	0.38891000	1.45559200
C	-3.60597300	1.37458900	0.45079500
C	-4.56204200	2.39222100	0.50984400
H	-4.56897000	3.15628800	-0.25941600
C	-5.50826600	2.45441600	1.54458000
C	-4.53135700	0.45559300	2.48838500
H	-4.50749100	-0.30341700	3.26346300
C	-2.53212300	-0.65587800	1.53289000
C	-5.48385100	1.46346300	2.54097900
H	-6.21137100	1.49488900	3.34449800
C	-1.74092800	-0.70168100	2.68893400
H	-1.87786600	0.06912200	3.43831000
C	-1.40058200	-2.64815800	0.74139700
H	-1.27572100	-3.42137300	-0.00705800
C	-0.78809500	-1.69104500	2.87474100
H	-0.17863300	-1.70857400	3.77028800
C	-0.60467800	-2.68182300	1.89768400
C	-0.06008100	3.92921600	-1.83804300
C	0.68377900	4.88269800	-1.87748900
H	1.38826700	5.68211600	-1.92029200
C	-6.47148000	3.50714800	1.58321600
C	0.35630700	-3.71556900	2.10785100
C	-5.63927800	-4.33079400	-1.95866800
C	-7.28521500	4.40167600	1.62022500

H	-8.00357400	5.18758400	1.64867600
C	1.17396400	-4.58184600	2.31904300
H	1.88492900	-5.35665700	2.48690200
C	-6.29173700	-5.34601700	-2.04363300
H	-6.86741900	-6.23916400	-2.11640800
C	2.89810000	-1.47132200	-0.49267900
C	2.91242800	0.85514700	-1.51421100
C	2.83559900	1.78114100	-0.45540100
C	3.26950000	3.09180400	-0.66042600
H	3.18640200	3.80850600	0.14850000
C	3.77901800	3.51160300	-1.89773400
C	3.42522800	1.27885500	-2.74934900
H	3.47970100	0.56280000	-3.56310400
C	2.37722800	-0.52512300	-1.39721200
C	3.85719300	2.58312900	-2.94823100
H	4.24739500	2.89491300	-3.91068500
C	1.35076300	-0.91394400	-2.26777100
H	0.95962300	-0.18331500	-2.96281500
C	2.37664000	-2.76762200	-0.48288300
H	2.77317100	-3.49354900	0.21539400
C	0.81824000	-2.19394000	-2.23957300
H	0.00609900	-2.46945200	-2.90338500
C	1.33202600	-3.14356800	-1.34181400
C	4.06490300	-1.16824300	0.38353300
C	2.76211200	0.52176500	1.74965600
C	2.20277100	1.45115900	0.85170000
C	1.05345000	2.15529600	1.21710100
H	0.63407400	2.87128200	0.52287600
C	0.43111100	1.94558500	2.45629500
C	2.16897100	0.35706900	3.00937100
H	2.61246800	-0.34649400	3.70659500
C	4.00272100	-0.23164300	1.43477800
C	1.02115200	1.05177000	3.36499300
H	0.56152100	0.89428100	4.33440300
C	5.12750100	-0.05403900	2.25326100
H	5.07298300	0.66649600	3.06309200
C	5.24219700	-1.89364900	0.18373500
H	5.28630600	-2.61893300	-0.62116600
C	6.29944800	-0.76777500	2.04277800
H	7.16366200	-0.61039300	2.67849100
C	6.37007900	-1.70371100	0.99747400
C	4.16506000	4.87222200	-2.08914200
C	4.46537200	6.03395000	-2.24943200
H	4.75652500	7.04878100	-2.39090800
C	-0.80785500	2.58674700	2.75939800
C	7.56332800	-2.45199100	0.76597800
C	0.79524200	-4.46539100	-1.31106200
C	-1.88567600	3.08533200	2.99447600
H	-2.85197100	3.49649200	3.17604900
C	8.57456500	-3.08706900	0.57176400
H	9.46496100	-3.64518700	0.39817900
C	0.31447100	-5.57546600	-1.29258300
H	-0.10157100	-6.55552300	-1.26750800

Calculation 5

Structure of TATP-Monomer

Method：#p opt=(cartesian,loose) b3lyp/6-31g(d,p) em=gd3bj

Total Energy (Hartree)= -1228.9494

Cartesian coordinates：

0 1

C	1.54684800	-0.49804100	-0.55385300
C	-0.55373900	0.49467400	-1.55490400
C	-1.54615200	0.49769400	-0.55493400
C	-2.59744100	1.41313100	-0.63410500
H	-3.36374000	1.40909400	0.13306100
C	-2.68154500	2.34152100	-1.68401600
C	-0.64962000	1.41210200	-2.61005300
H	0.11469600	1.40249400	-3.38045200

C	0.55520500	-0.49510200	-1.55461100
C	-1.68945300	2.33005700	-2.67889400
H	-1.74428400	3.04142100	-3.49528600
C	0.65208000	-1.41234500	-2.60978400
H	-0.11159800	-1.40280500	-3.38081100
C	2.59844400	-1.41315700	-0.63235200
H	3.36420900	-1.40899200	0.13534400
C	1.69222000	-2.33007000	-2.67794700
H	1.74781400	-3.04127600	-3.49442400
C	2.68356700	-2.34143600	-1.68236100
C	1.54629900	0.49753700	0.55466400
C	-0.55523900	-0.49511300	1.55431100
C	-1.54687100	-0.49791900	0.55353300
C	-2.59861100	-1.41287000	0.63204200
H	-3.36441400	-1.40857000	-0.13560900
C	-2.68394400	-2.34112400	1.68212500
C	-0.65229400	-1.41231000	2.60948000
H	0.11135900	-1.40288600	3.38053900
C	0.55383100	0.49454400	1.55459300
C	-1.69257200	-2.32991500	2.67765400
H	-1.74825500	-3.04107200	3.49417900
C	0.64972300	1.41189400	2.60978400
H	-0.11463000	1.40229300	3.38014900
C	2.59771700	1.41290000	0.63395500
H	3.36408200	1.40883900	-0.13314700
C	1.68962600	2.32974600	2.67874400
H	1.74444600	3.04102900	3.49520700
C	2.68181500	2.34124400	1.68396700
C	-3.75909600	3.27598200	-1.73828200
C	-4.67236000	4.06796300	-1.78421000
H	-5.47649600	4.76529700	-1.82464800
C	-3.76232000	-3.27469000	1.73586400
C	3.75942100	3.27563900	1.73880300
C	3.76162600	-3.27533900	-1.73613700
C	-4.67471100	-4.06763600	1.78358800
H	-5.47878500	-4.76497500	1.82457000
C	4.67262800	4.06769800	1.78519800
H	5.47676600	4.76505400	1.82608800
C	4.67459000	-4.06767600	-1.78198500
H	5.48080000	-4.76245700	-1.82403100

11. Reference

[1] M. J. Frisch, G. W. Trucks, H. B. Schlegel, G. E. Scuseria, M. A. Robb, J. R. Cheeseman, G. Scalmani, V. Barone, B. Mennucci, G. A. Petersson, H. Nakatsuji, M. Caricato, X. Li, H. P. Hratchian, A. F. Izmaylov, J. Bloino, G. Zheng, J. L. Sonnenberg, M. Hada, M. Ehara, K. Toyota, R. Fukuda, J. Hasegawa, M. Ishida, T. Nakajima, Y. Honda, O. Kitao, H. Nakai, T. Vreven, J. A. Montgomery, Jr., J. E. Peralta, F. Ogliaro, M. Bearpark, J. J. Heyd, E. Brothers, K. N. Kudin, V. N. Staroverov, T. Keith, R. Kobayashi, J. Normand, K. Raghavachari, A. Rendell, J. C. Burant, S. S. Iyengar, J. Tomasi, M. Cossi, N. Rega, J. M. Millam, M. Klene, J. E. Knox, J. B. Cross, V. Bakken, C. Adamo, J. Jaramillo, R. Gomperts, R. E. Stratmann, O. Yazyev, A. J. Austin, R. Cammi, C. Pomelli, J. W. Ochterski, R. L. Martin, K. Morokuma, V. G. Zakrzewski, G. A. Voth, P. Salvador, J. J. Dannenberg, S. Dapprich, A. D. Daniels, O. Farkas, J. B. Foresman, J. V. Ortiz, J. Cioslowski, and D. J. Fox, Gaussian, Inc., Wallingford CT, 2013.

[2] J. Tirado-Rives and W. L. Jorgensen, Performance of B3LYP Density Functional Methods for a Large Set of Organic Molecules, *J. Chem. Theory. Comput.* 2008, 4, 297-306.

[3] S. Grimme, J. Antony, S. Ehrlich and H. Krieg, A consistent and accurate ab initio parametrization of density functional dispersion correction (DFT-D) for the 94 elements H-Pu, *J. Chem. Phys.* 2010, 132, 154104.

[4] S. Grimme, S. Ehrlich and L. Goerigk, Effect of the damping function in dispersion corrected density functional theory, *J. Comput. Chem.* 2011, 32, 1456-1465.

[5] J. A. Rackers, Z. Wang, C. Lu, M. L. Laury, L. Lagardere, M. J. Schnieders, J. P. Piquemal, P. Ren, J. W. Ponder, Tinker 8: Software Tools for Molecular Design, *J. Chem. Theory. Comput.* 2018, 14, 5273-5289.

[6] J.-H. Lii and N. L. Allinger, Directional hydrogen bonding in the MM3 force field: II, *J. Comput. Chem.* 1998, 19, 1001-1016.

[7] F. Santoro, R. Improta, A. Lami, J. Bloino, and V. Barone, J. Chem. Phys. 2007, 126, 084509.

[8] S. Liu, Y. Gao, K. Zhang, S. Liu, H. Lan, L. Lin, C.-K. Wang, J. Fan, and Y. Song, Phys. Chem. Chem. Phys. 2022, 24, 22905.

[9] B. Knapp, N. Lederer, U. Omasits, and W. Schreiner, J. Comput. Chem. 2010, 31, 2868.

[10] W. Zhou, Y. C. Liu, G. J. Liu, Y. Zhang, G. L. Feng, G. W. Xing, Glycosylated AIE-active Red Light-triggered Photocage with Precisely Tumor Targeting Capability for Synergistic Type I Photodynamic Therapy and CPT Chemotherapy, *Angew. Chem. Int. Ed.* 2025, 64, e202413350.

[11] H. Jiang, Y. Zhang, D. S. Chen, B. Zhou, Y. H. Zhang, An Approach to Tetraphenylenes via Pd-Catalyzed C–H Functionalization, *Org. Lett.* 2016, 18, 2032-2035.
